# Supramolecular Self‐Assembly of Organic Cu(I) Iodides as Green and Recyclable Paper‐Based Customizable Large‐Area Flexible Film for Plant Grow Lighting and Plant Root X‐Ray Imaging

**DOI:** 10.1002/advs.202513081

**Published:** 2025-10-05

**Authors:** Shengji Yuan, Hui Peng, Hairui Zhang, Ou Xu, Fei Wang, Wenchao Yang, Zhentao Du, Bingsuo Zou

**Affiliations:** ^1^ Guangxi Key Laboratory of Processing for Non‐ferrous Metals and Featured Materials and School of Resources Environment and Materials Guangxi University Nanning 530004 China

**Keywords:** green and recyclable, organic Cu(I) iodides, plant growth lighting, supramolecular assembly, X‐ray imaging

## Abstract

Regarding global energy scarcity issues, it is imperative to establish sustainable plant production systems to promote smart agriculture. Addressing the critical demands of plant lighting and X‐ray imaging in smart agriculture, a recyclable luminescent flexible film is developed on a filter paper and organic Cu(I) metal iodide, and establishes a bifunctional platform integrating plant growth lighting and plant root X‐ray imaging. Specifically, [Ca(15‐crown‐5)_2_]Cu_4_I_6_·2C_3_H_7_NO·H_2_O and [Ca_2_(18‐crown‐6)_4_]Cu_4_I_8_·6H_2_O are synthesized through supramolecular assembly, which show yellow emission with the near‐unity luminous efficiency under 450 nm excitation. Moreover, organic Cu(I) iodides exhibit bright X‐ray radioluminescence with a maximum light yield of 110200 photons per MeV. Subsequently, a paper‐based customizable large‐area flexible film is prepared in situ through “ancient cloth dyeing process”, and demonstrates its application in single‐component white light emitting diode and X‐ray imaging. Combined white light and X‐ray image fusion as well as multiangle imaging, the flexible film in the application of 3D image reconstruction is demonstrated. Furthermore, the flexible film can be recycled and reused in a N, N‐dimethylformamide solution while maintaining excellent stability. Considering that the flexible film can be degraded by microorganisms in natural soil into plant nutrients and participate in the carbon loop, it is conducive to achieving carbon neutrality.

## Graphical abstract

Organic Cu(I) iodides with efficient broadband photoluminescence and radioluminescence are reported. Based on “ancient cloth dyeing process”, a paper‐based flexible film is prepared in situ on cellulose, and demonstrates its application in plant growth lighting and plant root X‐ray imaging. Moreover, the film has the characteristics of recyclability, biodegradability, and sustainability, so it has great application potential in smart agriculture.

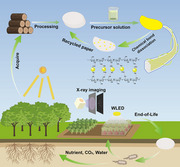

## 1 Introduction

According to the United Nations Sustainable Development Goals report for 2024, ≈7.5% of the global population faced hunger in 2019, and this figure has risen to 9.1% by 2023. This urgent situation requires the establishment of sustainable agricultural production systems to substantially increase crop yields. Smart agriculture has emerged as a critical breakthrough in addressing food security challenges through next‐generation flexible electronic sensors, agricultural monitoring systems, and Internet of Things platforms to optimize variability and uncertainty within agricultural systems to enhance productivity.^[^ ^1^, ^2^, ^3^ ^]^ In smart agricultural systems, plant lighting and plant root X‐ray imaging play a crucial role because lighting directly influences photosynthesis, while X‐ray imaging can monitor the health status of plants in real time.^[^ ^4^, ^5^ ^]^ As a key component of lighting and X‐ray imaging, the photophysical properties of luminescent materials are closely related to the advanced level of smart agricultural system. Therefore, it is crucial to develop luminescent materials with efficient emission.

Recently, a series of lead‐free metal halide luminescent materials have been developed, which show efficient broadband emission and negligible self‐absorption, thus showing great application potential in advanced optoelectronic devices.^[^ ^6^, ^7^, ^8^ ^]^ Among numerous candidates, Cu(I)‐based metal halides stand out due to their non‐toxic and low‐cost characteristics. At present, many all‐inorganic Cu(I)‐based metal halides (e.g., CsCu_2_X_3_ (X = Cl, Br, I),^[^ ^9^ ^]^ Cs_5_Cu_3_Cl_6_I_2_,^[^ ^10^ ^]^ Rb_2_CuCl_3_^[^ ^11^ ^]^) and organic hybrid Cu(I)‐based metal halides (e.g., (MA)_4_Cu_2_Br_6_,^[^ ^12^ ^]^ (TBA)_2_Cu_2_I_4_,^[^ ^13^ ^]^ (TBP)_2_Cu_4_Br_6_,^[^ ^14^ ^]^ α‐(ETPA)_2_Cu_2_I_4_,^[^ ^15^ ^]^ [LB]_2_Cu_4_I_6_^[^ ^16^ ^]^) have been reported. Obviously, organic hybrid Cu(I)‐based metal halides have richer optical properties due to the diversity of organic ligands, which makes them show great prospects in the application of white‐light‐emitting diodes (WLEDs) and X‐ray scintillators. For example, [(C_3_H_7_)_4_N]_2_Cu_2_I_4_ exhibits efficient white emission with a PLQY of 91.9% and a color rendering index (CRI) of 92.2.^[^ ^17^ ^]^ Parallelly, [BzTPP]_2_Cu_2_I_4_ emits bright radioluminescence (RL) with a light yield of 27706 photons per MeV and a detection limit of 352 nGy_air_/s, and the corresponding X‐ray imaging spatial resolution of the [BzTPP]_2_Cu_2_I_4_/polyethylene pyrrolidone film is 4.928 lp mm^−1^.^[^ ^18^ ^]^ Although some progresses have been made in the study of organic hybrid Cu(I)‐based metal halides, there are still some bottlenecks in their application in advanced optoelectronic devices. First, the currently reported organic hybrid Cu(I)‐based metal halides mainly introduce various multifunctional organic ligands aimlessly into inorganic lattices, lacking rational design principles for designing organic hybrid Cu(I)‐based metal halides with high PLQY and high light yield. Second, the excitation bands of most organic hybrid Cu(I)‐based metal halides are located in the ultraviolet (UV) region. However, the efficiency of UV chips is far lower than that of blue LED chips,^[^ ^19^ ^]^ which results in poor optoelectronic performance of the as‐fabricated WLEDs. Third, the preparation of flexible X‐ray scintillation screens mainly involves integrating organic hybrid Cu(I)‐based metal halide powders into polymers, such as polyvinylidene difluoride (PVDF), polydimethylsiloxane (PDMS), and poly(methyl methacrylate) (PMMA).^[^ ^20^, ^21^ ^]^ However, they typically exhibit poor X‐ray imaging spatial resolution due to the serious light scattering caused by uneven dispersion. In addition, polymers are difficult to degrade, which poses a threat to the environment.

Crown ethers (e.g., 15‐crown‐5, 18‐crown‐6) are functional ligands with rich coordination properties, which have strong metal chelating ability. Moreover, crown ethers can bind to alkali metal cations (e.g., Na^+^, K^+^, Rb^+^, Cs^+^) or alkaline‐earth metal cations (e.g., Ba^2+^, Sr^2+^, Ca^2+^, Mg^2+^) to form supramolecular cations.^[^ ^22^, ^23^, ^24^ ^]^ Benefiting from the strong supramolecular interaction between metal cations and crown ethers, the rigidity of the crystal structure can be effectively increased, thereby suppressing non‐radiative transitions and bringing rich optical properties.^[^ ^25^ ^]^ As a renewable material, cellulose paper possesses the advantages of lightweight, biocompatibility, excellent processability, and suitability for large‐area fabrication, which meets the needs of green electronics.^[^ ^26^, ^27^, ^28^ ^]^ In addition, cellulose paper is a flexible thin layer material with 3D porous network structure formed by interweaving fibers, which makes it an excellent carrier of luminescent materials, thus preparing a large‐area flexible film.

**Scheme 1:**
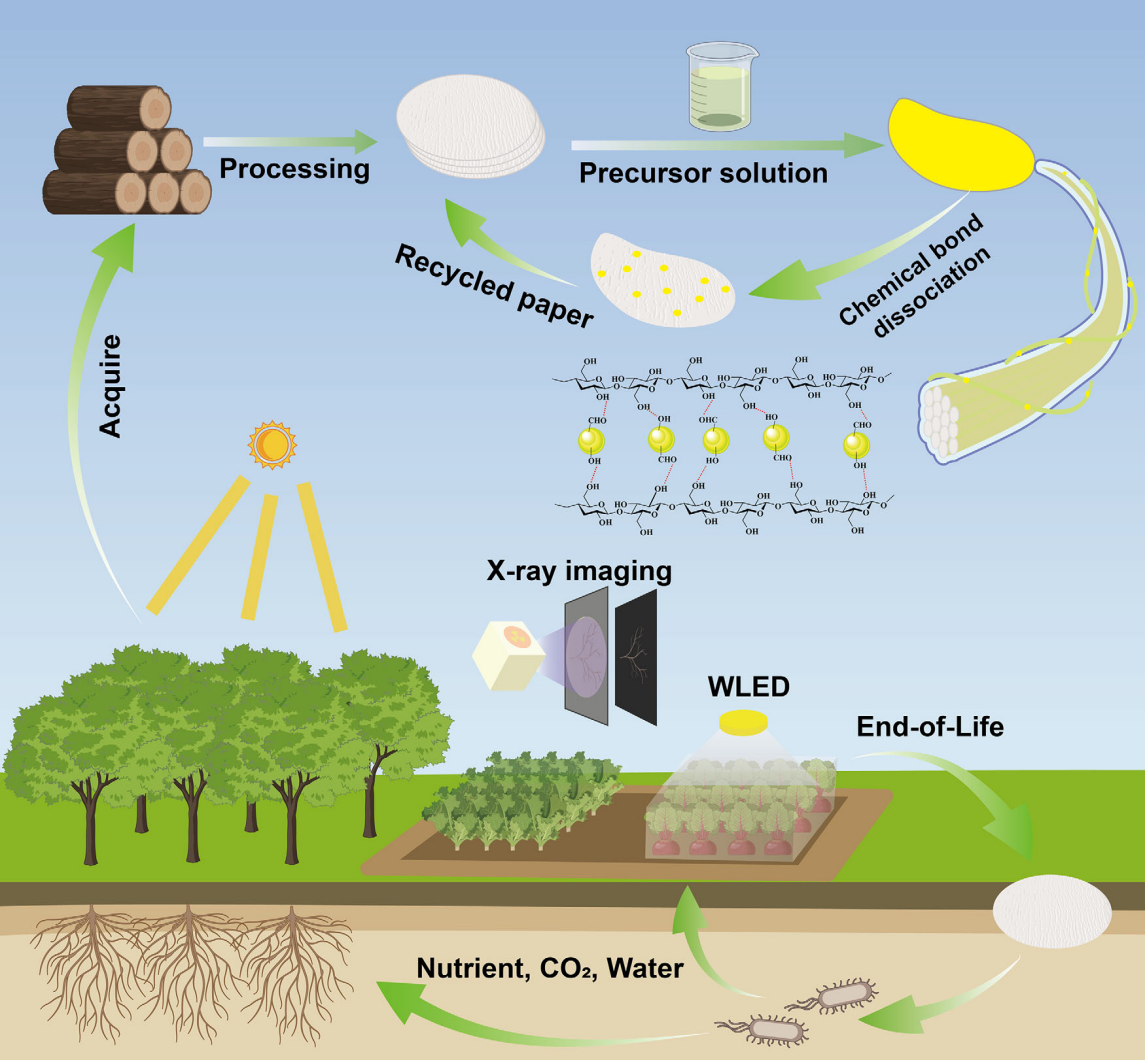
Schematic diagram of the fabrication, degradation, and recycling of **Compound‐I**@paper film based on filter paper and **Compound‐I** microcrystals, as well as their applications in plant grow lighting and plant root X‐ray imaging.

In this work, we synthesized two 0D organic hybrid Cu(I)‐based metal iodides with efficient broadband emission using supramolecular self‐assembly strategy. Under 450 nm excitation, [Ca(15‐crown‐5)_2_]Cu_4_I_6_·2C_3_H_7_NO·H_2_O (**Compound‐I**) and [Ca_2_(18‐crown‐6)_4_]_2_Cu_4_I_8_·6H_2_O (**Compound‐II**) exhibit efficient broadband yellow emission with PLQYs of 99.0% and 90.1%, respectively. Interestingly, **Compound‐II** can undergo reversible structural transformation with [Ca(18‐crown‐6)]Cu_5_I_7_·3H_2_O (**Compound‐III**) under heat or methanol treatment, accompanied by reversible photoluminescence (PL) switching between yellow emission and non‐emission. Compared with **Compound‐II**, **Compound‐I** shows remarkable X‐ray scintillation properties with an ultra‐high light yield of 110200 photons per MeV and an ultra‐low detection limit of 65 nGy_air_/s. Based on “ancient cloth dyeing process” and using a common filter paper as the carrier, a large‐area flexible paper‐based customizable film was successfully prepared by uniformly precipitating **Compound‐I** microcrystals in situ on cellulose (**Scheme** **1**). Afterwards, using the **Compound‐I**@paper film as the emission layer of the single‐component WLED and the X‐ray scintillation screen, a luminous efficiency of 114.5 lm W^−1^ and a spatial resolution of 21.6 lp mm^−1^ were achieved, respectively. The 3D model of irregular iron sheet wrapped in an opaque cardboard box was reconstructed by combining white light and X‐ray image fusion as well as multiangle imaging. Moreover, the end‐of‐life **Compound‐I**@paper film can be recycled by dissolving **Compound‐I** microcrystals in N, N‐dimethylformamide (DMF) to recover filter paper frame and **Compound‐I** solution for reuse, paving a new path for circular economy. More importantly, the **Compound‐I**@paper film has good biodegradability. The cellulose in it can be naturally degraded by water and microorganisms in the soil and can be used as plant fertilizer, which is conducive to achieving ecological closed loop and carbon neutrality.

## 2 Results and Discussion

**Figure 1:**
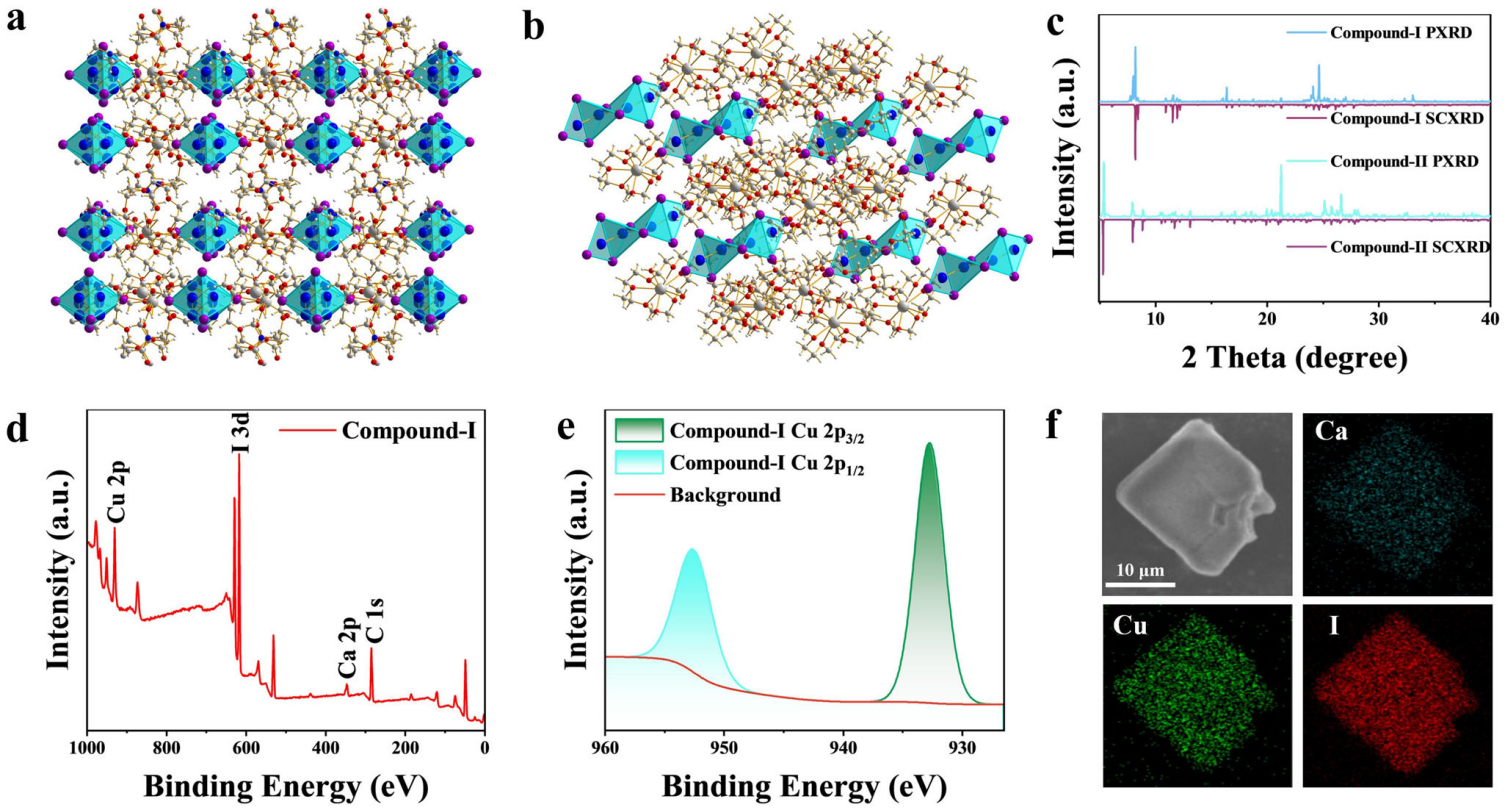
Crystal structures of a) **Compound‐I** and b) **Compound‐II**. c) PXRD patterns of organic Cu(I) iodides and the corresponding simulated results. d) XPS spectrum of **Compound‐I** and e) the corresponding fine XPS spectrum of Cu 2p. f) Scanning electron microscopy (SEM) image of **Compound‐I** and the corresponding element mapping results.

Bulk crystals of **Compound‐I** and **Compound‐II** were synthesized by solution method. In both organic Cu(I) iodides, **Compound‐I** crystallizes in the monoclinic system with *P*2_1_/*c* symmetry, while **Compound‐II** adopts a triclinic system with *P*‐1 symmetry. Detailed crystallographic data are given in Table S1 (Supporting Information). Parallelly, Ca^2+^ ions can coordinate with crown to form supramolecular cations of [Ca(15‐crown‐5)_2_]^2+^ for **Compound‐I** and [Ca(18‐crown‐6)_2_]^2+^ for **Compound‐II**, respectively. In addition, C_3_H_7_NO (DMF) and H_2_O enter the lattice of **Compound‐I**, while H_2_O enters the lattice of **Compound‐II**. Particularly, all inorganic clusters of [Cu_4_I_6_]^2−^ with rhombus structure in **Compound‐I** and [Cu_4_I_8_]^4−^ with edge‐sharing dimer cluster structure in **Compound‐II** are uniformly distributed within the host framework formed by supramolecular cations, resulting in unique 0D structure (**Figure** **1**a,b). The shortest Cu‐Cu distances are 1.88 Å in [Cu_4_I_6_]^2−^ and 2.74 Å in [Cu_4_I_8_]^4−^ clusters, which is smaller than the sum of the van der Waals radii of Cu^+^ (2.80 Å). Thus, there are strong metal‐metal bonding interaction in both organic hybrid Cu(I)‐based compounds.^[^ ^29^ ^]^

Figure 1c gives the powder X‐ray diffraction (PXRD) patterns of organic Cu(I) iodides, which is similar to the simulated single crystal XRD (SCXRD) results, indicating that the as‐synthesized compounds have high phase purity. Figure 1d and Figure S1a (Supporting Information) depict the X‐ray photoelectron spectroscopy (XPS) spectra of **Compound‐I** and **Compound‐II**, respectively, and characteristic peaks of Cu, Ca, and I can be observed. Moreover, the fine XPS spectra of Cu 2p show two distinct peaks at 932.5 eV for Cu 2p_3/2_ and 952.7 eV for Cu 2p_1/2_ (Figure 1e; Figure S1b, Supporting Information), which demonstrates Cu is in the +1 valence state in both compounds. The element mapping results show that Cu, Ca, and I have a homogeneous distribution on the crystal surface (Figure 1f; Figure S2a, Supporting Information). Table S2 (Supporting Information) lists the energy dispersive spectrometer (EDS) results of both organic Cu(I) iodides, and the molar ratio of Ca:Cu:I is in line with the experimental SCXRD results.

**Figure 2:**
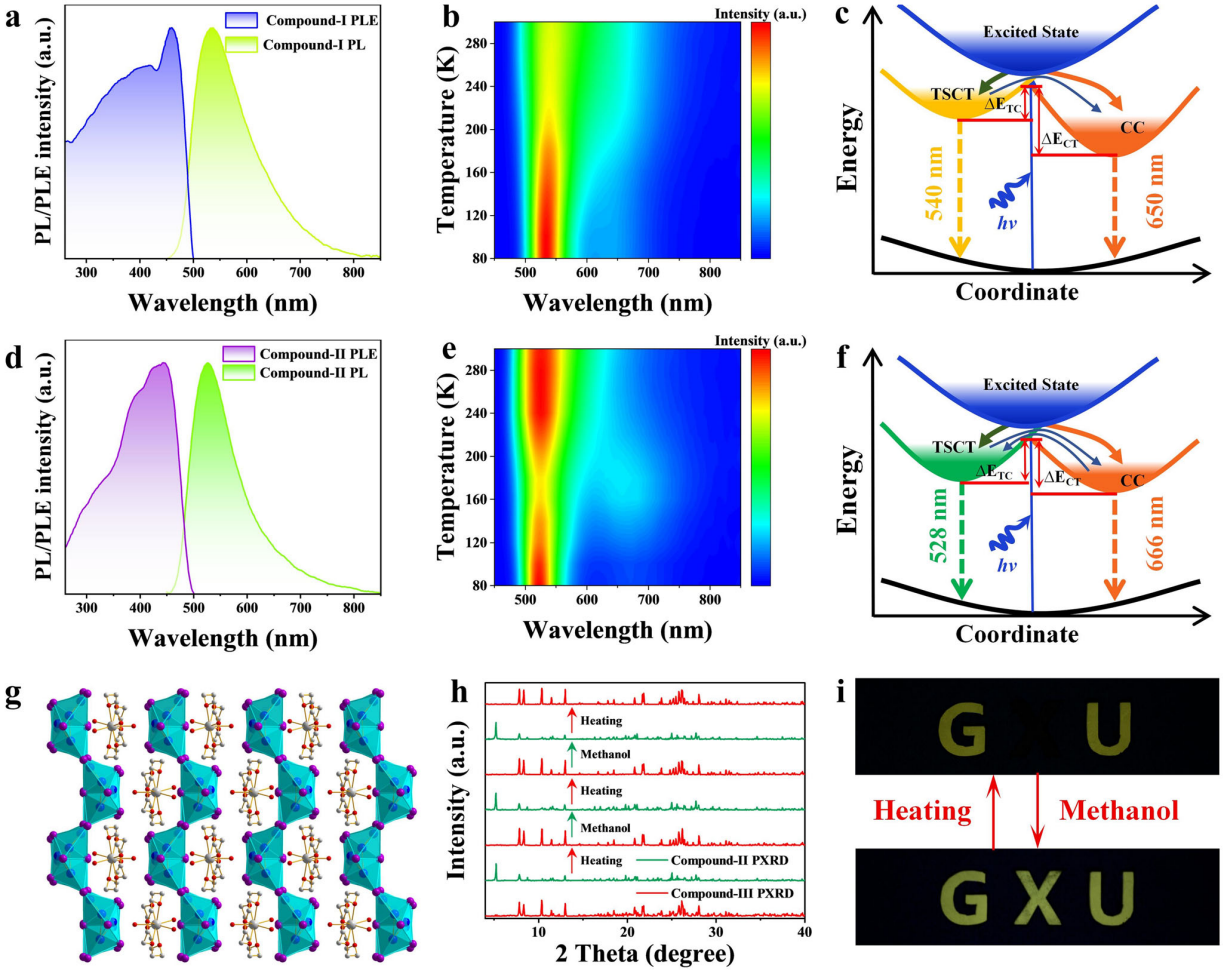
a) PL and PLE spectra of **Compound‐I**. b) Temperature‐dependent PL spectra of **Compound‐I**. c) Proposed photophysical processes of **Compound‐I**. d) PL and PLE spectra of **Compound‐II**. e) Temperature‐dependent PL spectra of **Compound‐II**. f) Proposed photophysical processes of **Compound‐II**. g) Crystal structure of **Compound‐III**. h) PXRD patterns of **Compound‐II** after treatment with heating or methanol. i) Photographs of “GXU” pattern under 365 nm irradiation.

The optical properties of **Compound‐I** and **Compound‐II** were investigated in detailed. At room temperature, both organic Cu(I) iodides emit broadband emission, which is composed of a high‐energy emission band (540 nm for **Compound‐I** and 528 nm for **Compound‐II**) and a tail in the lower‐energy region (**Figure** **2**a,d), and the corresponding CIE color coordinates are (0.39, 0.55) and (0.35, 0.57), respectively (Figure S3, Supporting Information). In the excitation (PLE) spectra, both organic Cu(I) iodides exhibit ultrabroad excitation bands that can cover the ultraviolet (UV) and blue regions, which can be attributed to the strong cation‐cation bonding within the inorganic cuprous iodide clusters.^[^ ^30^ ^]^ Moreover, there is a large Stokes of 90 nm for **Compound‐I** and 83 nm for **Compound‐II**, which indicates that they have negligible self‐absorption. Particularly, **Compound‐I** and **Compound‐II** have ultra‐high PLQY of 99.0% and 90.1% under 450 nm excitation (Figure S4, Supporting Information). There near‐unity PLQY of organic Cu(I) iodides can be assigned to the strong supramolecular interaction can improve the structure rigidity, thereby boosting the radiative transition.^[^ ^31^ ^]^ Combined with the room temperature PL decay lifetime of 2.01 µs for **Compound‐I** and 1.81 µs **Compound‐II** (Table S3, Supporting Information), the non‐radiative decay rates of **Compound‐I** and **Compound‐II** are calculated to be 0.49 × 10^4^ s^−1^ and 5.41 × 10^4^ s^−1^, respectively. The drastic suppression of non‐radiative transition in **Compound‐I** can ensure that it has higher PLQY.^[^ ^22^ ^]^ Figure S5 (Supporting Information) shows the absorption spectra of **Compound‐I** and **Compound‐II**, and the corresponding optical absorption edge at 485 and 480 nm, respectively. Moreover, the bandgap values of both compounds were determined to be 2.53 and 2.57 eV through the Tauc plot.

To further elucidate the luminescence mechanism, the temperature‐dependent PL spectra of Cu(I)‐based metal halides were measured within 80–300 K (Figure 2b,e). Compared with the room temperature PL spectra, the low temperature PL spectra were split into two different emission bands. Figure S6 (Supporting Information) shows the temperature‐dependent PL decay curves of **Compound‐I** and **Compound‐II**, respectively. Compared with 300 K, the PL decay lifetime of the corresponding emission band increases significantly at 80 K. This result should be attributed to the suppression of thermally activated nonradiative recombination at low temperature. Furthermore, the dual‐emission bands at 80 K exhibit different PL lifetimes, demonstrating that they stem from different excited states. Subsequently, the band structures and density of states (DOS) of **Compound‐I** and **Compound‐II** were calculated using density functional theory (DFT). In Figure S7a,b, both organic Cu(I) iodides show the direct bandgap of 1.10 eV for **Compound‐I** (Figure S7a, Supporting Information) and 1.51 eV for **Compound‐II** (Figure S7b, Supporting Information), which is much lower than the experimental bandgap values due to the acknowledged Perdew−Burke−Ernzerhof function error.^[^ ^32^ ^]^ In the DOS, the band edges of organic Cu(I) iodides are mainly composed of organic ligand and inorganic cuprous iodide cluster (Figure S7c,d). More particularly, there is a short Cu‐Cu distance in both organic Cu(I) iodides. Hence, the dual‐emission band in our organic Cu(I) iodides can be assigned to metal‐to‐ligand or halide‐to‐ligand charge transfer (MLCT/HLCT) and cluster‐centered (CC) excited state.^[^ ^33^, ^34^ ^]^ Considering that organic Cu(I) iodides are ionic species, in which there are only counter cations rather than organic ligands, the through‐space charge transfer (TSCT) should be used instead of the MLCT/HLCT.^[^ ^35^ ^]^ Thus, the high‐energy and low‐energy emission bands in both organic Cu(I) iodides should originate from the radiative relaxation of TSCT and CC excited states, respectively. In addition, there are two different energy barriers, namely, the energy barrier from TSCT to CC state (ΔE_TC_) and the energy barrier from CC to TSCT state (ΔE_CT_). Generally, the PL intensity of organic Cu(I)‐based metal halides decreases gradually with the increase of temperature due to the influence of PL thermal quenching,^[^ ^36^ ^]^ as observed in **Compound‐I** (Figure 2b). By contrast, the PL intensity of the low‐energy emission band of **Compound‐II** exhibits abnormal anti‐thermal quenching behavior at low‐temperature zone, which is due to the energy barrier of ΔE_TC_ in **Compound‐II** is greater than that in **Compound‐I**. Therefore, only at high temperatures can the photogenerated excitons in the TSCT state of **Compound‐II** overcome the energy barrier and reach the CC state with the aid of thermal energy, effectively compensating for the PL quenching of the low‐energy PL band.^[^ ^37^ ^]^ With further increasing of temperature, the PL intensity of low‐energy emission band of **Compound‐II** begins to decrease, while the PL intensity of high‐energy emission band is enhanced, which should be due to the thermal‐enhanced energy transfer from the low‐energy CC state to the high‐energy TSCT state,^[^ ^38^ ^]^ thus the high‐energy emission band exhibits the anti‐thermal quenching behavior at high‐temperature zone.

Figure 2c,f summarizes the photophysical mechanism of organic Cu(I) iodides. Upon photoexcitation, electrons can transition from the ground state to the excited state, thereby generating free excitons. Then, the free excitons can relax to TSCT and CC states through intersystem crossing or internal conversion, resulting in high‐energy and low‐energy PL bands, respectively.^[^ ^39^ ^]^

Also noteworthy, **Compound‐I** has remarkable stability. When it was stored in the air for 150 days, its PLQY remained at a high level (Figure S8a, Supporting Information), and the corresponding PXPD pattern also showed a similar profile to the pristine one (Figure S8b, Supporting Information). Under 450 nm irradiation for 7200 s, the PL intensity shows only slight attenuation (Figure S8c, Supporting Information). Hence, **Compound‐I** has a great application potential in solid‐state lighting and X‐ray scintillator.

By contrast, we unexpectedly found that the yellow emission of **Compound‐II** can be transformed into non‐emission after heating treatment (Figure S9a, Supporting Information), but this phenomenon does not exist in **Compound‐I**. To explore the luminescence transition mechanism of **Compound‐II**, the structure of the crystal with non‐emission was measured and it was found that it transformed into [Ca(18‐crown‐6)]Cu_5_I_7_·3H_2_O (**Compound‐III**). In Figure 2g, **Compound‐III** shows a zigzag 1D chain structure, where the neighboring [Cu_5_I_7_]^2−^ are connected via edge sharing. Meanwhile, this compound belongs to monoclinic space group Pbmc, and the related crystallographic parameters are great different from **Compound‐II** (Table S1, Supporting Information). Moreover, the XPS spectra and SEM image of **Compound‐III** are given in Figures S1c,d and S2b (Supporting Information), respectively. In particular, the non‐emission of **Compound‐III** can return to yellow emission after treatment by methanol, and the PL spectral profile is similar to that of **Compound‐II** (Figure S9b, Supporting Information). This result illustrates that the PL switching observed in **Compound‐II** and **Compound‐III** is reversible,^[^ ^40^ ^]^ which was demonstrated by the PXRD patterns of **Compound‐II** treated with heating or methanol (Figure 2h). Furthermore, the reversible PL switching shows remarkable stability, and the PL intensity remains basically unchanged after twenty consecutive cycles (Figure S9c, Supporting Information).

Based on the reversible PL switching characteristics of organic Cu(I) iodides, we then demonstrated its application in fluorescent anti‐counterfeiting. In Figure 2i, **Compound‐I** powders were filled into “GU”, while **Compound‐II** powders were filled into “X”. Under 365 nm irradiation, “GXU” shines bright yellow emission. After heating treatment, “GU” exhibits yellow emission, while “X” shows non‐emission. As we expected, the yellow emission of “GXU” can be observed after methanol treatment. Hence, this special dual‐mode fluorescence anti‐counterfeiting can effectively ensure the safety of products. In addition, a complex Morse code information encryption system was further constructed, as shown in Figure S10 (Supporting Information).

**Figure 3:**
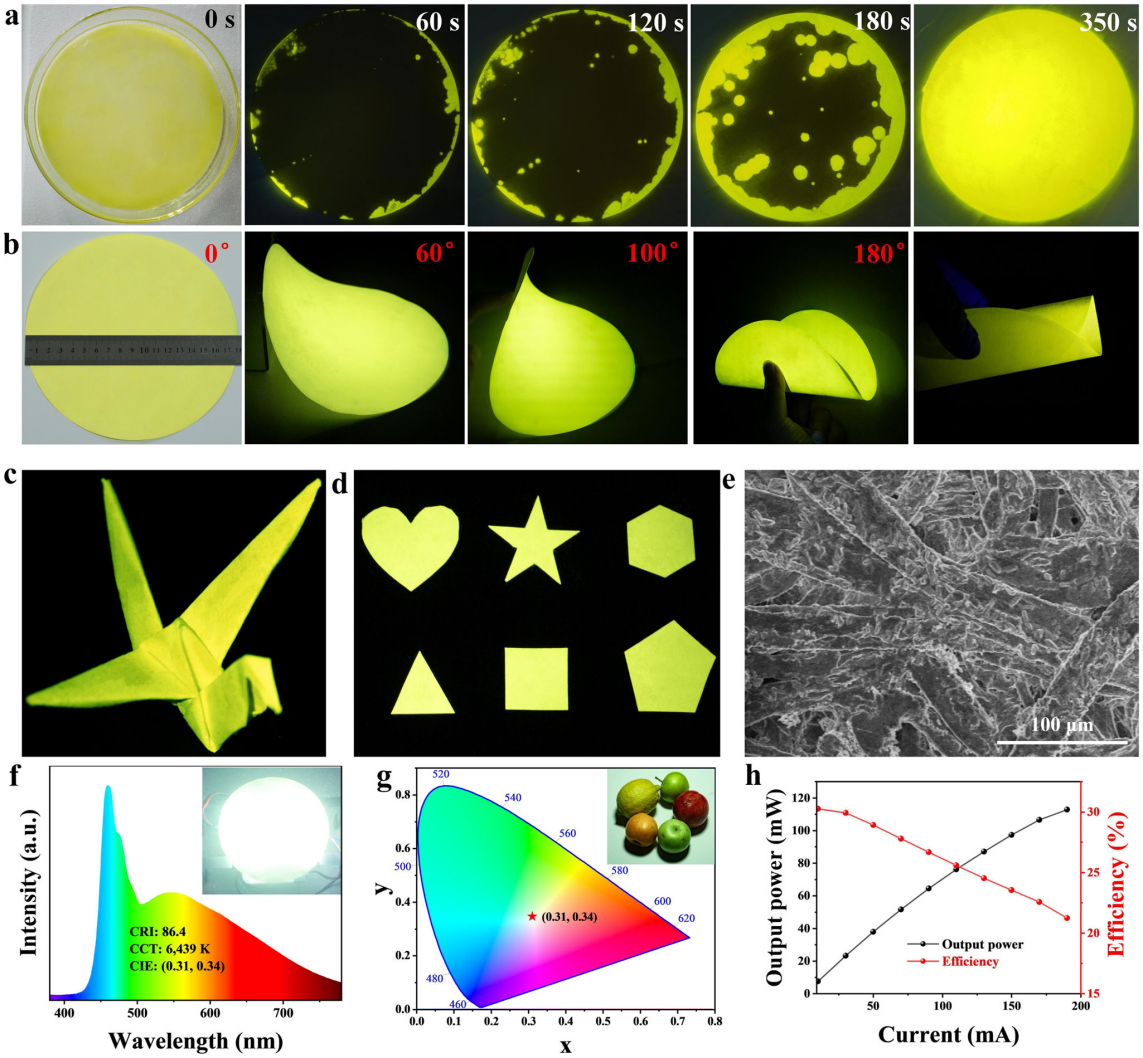
a) Optical images of filter paper embedded with **Compound‐I** microcrystals at different times. b) Optical images of **Compound‐I**@paper film with different bending angles. c) Optical image of “Crane” prepared by **Compound‐I**@paper film under 365 nm irradiation. d) Optical images of **Compound‐I**@paper film with arbitrary customizable shape prepared by cutting. e) Top‐view SEM image of **Compound‐I**@paper. f) Emission spectrum of **Compound‐I**@paper based WLED under 460 nm LED array excitation. g) CIE coordinate of WLED, and the inset shows the fruits under WLED irradiation. h) Output power and photoelectric conversion efficiency of WLED.

Considering the excellent solution processability, highly efficient broadband emission, and excellent stability of **Compound‐I**, we have prepared a large‐area flexible film using **Compound‐I** and demonstrated its application in advanced optoelectronic devices. Generally, the reported metal halide luminescent films use polymers as carriers, such as PVDF, PDMS, and PMMA,^[^ ^10^, ^41^, ^42^ ^]^ which exhibit serious drawbacks such as high cost, complex production processes, and difficulty in degradation. Selecting an appropriate carrier is crucial for practical applications in green and recyclable smart agriculture. Among numerous candidates, paper stands out as particularly advantageous due to its low cost, easy availability, biodegradability, and remarkable flexibility. Therefore, we selected filter paper as the carrier and prepared **Compound‐I**@paper flexible film with a thickness of 100 µm through the “ancient cloth dyeing process”. First, 15‐crown‐5, CuI, and CaI_2_ were dissolved in DMF solution, and then the filter paper was fully immersed in the precursor solution. The precursor ions can gradually diffuse into cellulose molecular chains based on capillary effects. To investigate the in‐situ formation processes of luminescent film, the optical photographs of **Compound‐I**@paper were recorded at different times (**Figure** **3**a). When the soaked filter paper is transferred to a hot stage at 40 °C, a bright yellow emission appears from the edge of the filter paper and gradually spreads as the DMF solvent evaporates, eventually uniformly covering the entire filter paper. This can be attributed to the diffusion of precursor ions and the effective coordination between organic Cu(I) iodide and cellulose molecular chains. Consequently, **Compound‐I**@paper with an area of 254.5 cm^2^ was successfully prepared within 350 s. Figure S11a (Supporting Information) shows the emission spectrum of **Compound‐I**@paper, and its spectral characteristic is consistent with **Compound‐I**. In Figure 3b, **Compound‐I**@paper exhibits excellent flexibility, which can be folded into any angle without damage. After 100 consecutive folds, the emission intensity of **Compound‐I**@paper remains at a high level (Figure S11b, Supporting Information), which suggests that the **Compound‐I** microcrystals have excellent adhesion on filter paper. Moreover, **Compound‐I**@paper shows a foldable characteristic (Figure 3c) and can be cut into any customizable shape (Figure 3d), which makes it perfectly match the target object. Generally, filter paper is composed of numerous micro‐size cellulose nanofibrils (Figure S12, Supporting Information), which can provide abundant nucleation sites for the growth of the **Compound‐I** microcrystals. Moreover, due to the presence of abundant ‐OH polar functional groups in the cellulose molecular chain, they can react with ‐OH in water molecules and ‐CHO in DMF in **Compound‐I**, thereby promoting the uniform crystallization on filter paper (Scheme 1a), as confirmed by Fourier‐transform infrared spectroscopy (Figure S13, Supporting Information). Moreover, we also prepared **Compound‐I**@paper films at 20 and 60 °C, respectively (Figure S14a,d, Supporting Information), and the results show that **Compound‐I**@paper scintillation screen prepared at 40 °C has higher quality. To illustrate this, the top‐SEM images of **Compound‐I**@paper scintillation screen were collected. At low temperature of 20 °C, **Compound‐I** microcrystals exhibit a large size (Figure S14b, Supporting Information), resulting in uneven dispersion on the filter paper. At high temperature of 60 °C, the nucleation rate of crystals is too fast, resulting in the formation of smaller microcrystals accompanied by agglomeration (Figure S14e, Supporting Information). By contrast, **Compound‐I** microcrystals on the scintillation screen prepared at 40 °C show uniform dispersion (Figure 3e) with an average particle size of 7.01 µm (Figure S15, Supporting Information), thus obtaining a high‐quality flexible film.

Generally, WLEDs are employed as plant light source in smart agriculture for their superior luminous efficiency, high stability, long life, and high degree of spectral matching with plant absorption spectra, which is conducive to plants to achieve photosynthesis. In this work, benefiting from **Compound‐I** emits efficient yellow emission under blue excitation, a single‐component WLED can be fabricated by coating **Compound‐I**@paper film on a 460 nm LED array (Figure S16a,b, Supporting Information). The as‐fabricated device emits bright white emission after operation (Figure 3f, inset), and the corresponding broadband white emission and absorption required for plant growth lighting overlap well (Figure 3f), which will be beneficial for promoting plant growth (Figure S17, Supporting Information).^[^ ^43^ ^]^ The CIE color coordinate of the WLED is (0.31, 0.34), and the corresponding correlated color temperature (CCT) is 6439 K (Figure 3f). Moreover, the device has a high CRI of 86.4, far higher than that of fluorescent lamp with a CRI of 72,^[^ ^44^ ^]^ which makes the device have good color reproduction (Figure 3g, inset). Notably, the WLED has an ultra‐high luminous efficiency of 114.5 lm W^−1^, which is currently the highest value reported for metal halides (Table S4, Supporting Information). Figure 3h shows the output power and photoelectric conversion efficiency of the WLED under various drive currents. Clearly, the output power increases gradually as the increase of drive current. By contrast, the photoelectric conversion efficiency decreases gradually under high driving current, which should be attributed to the “efficiency droop” of the blue LED.^[^ ^45^ ^]^ Moreover, the WLED has remarkable operational stability. In Figure S16c (Supporting Information), when the WLED continues to operate for 420 min, the emission intensity remains at 80%. Compared with the traditional WLED made of polymer encapsulated oxide phosphors or lead halide perovskites, our WLED based on **Compound‐I**@paper film has the advantages of environmental protection, high integration, biodegradability, and recyclability, which can build a multifunctional platform for sustainable smart agriculture (Table S5, Supporting Information).

**Figure 4:**
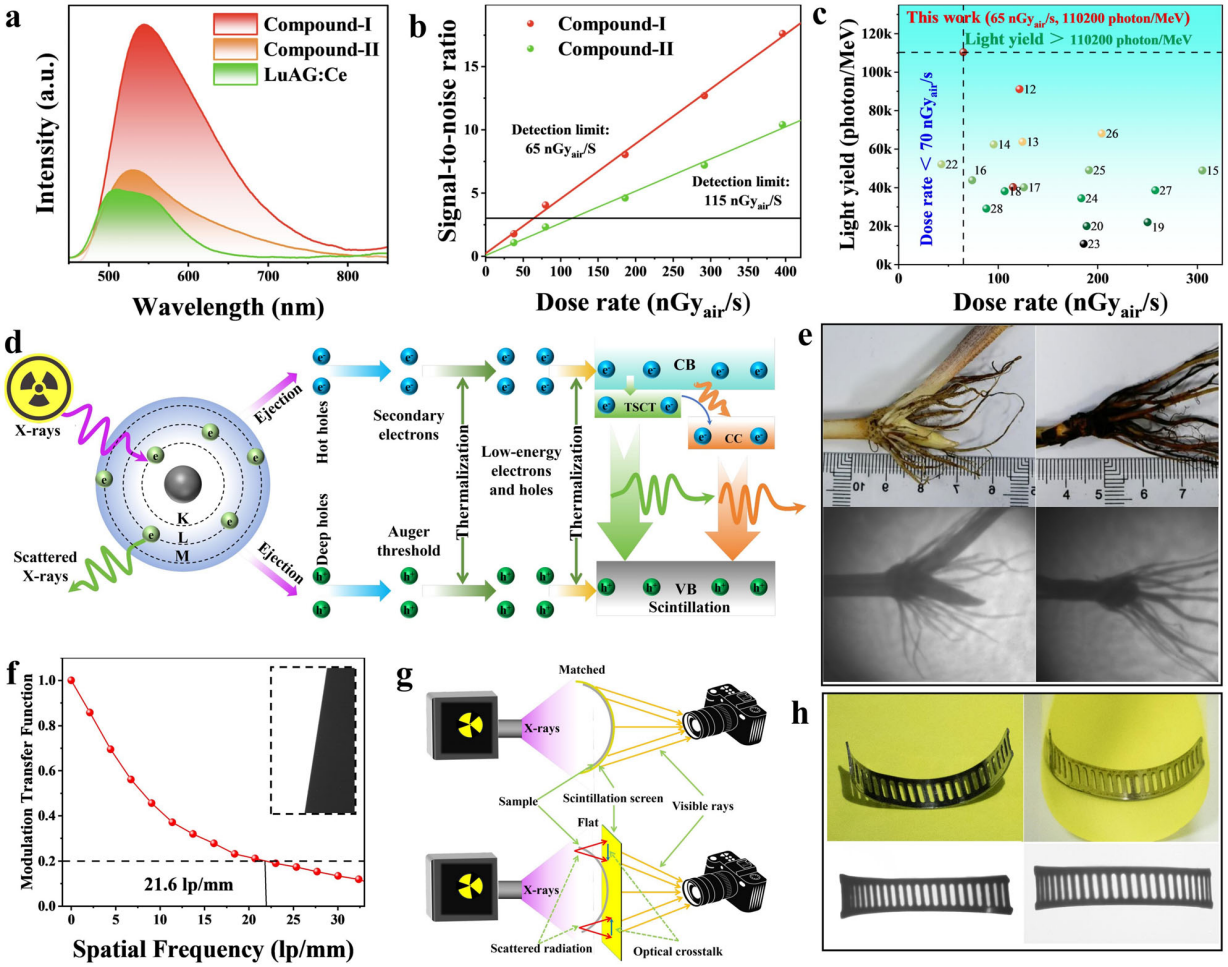
a) RL spectra of **Compound‐I**, **Compound‐II**, and LuAG:Ce under X‐ray excitation (dose rate, 0.19 mGy_air_/s). b) Linear fitting between signal‐to‐noise ratio and dose rate. c) Dose rate versus light yield for previously reported lead‐free metal halide scintillators (inserted numbers represent the references in Table S6, Supporting Information). d) Proposed RL mechanism of organic Cu(I) iodides. e) Photographs of X‐ray images. f) Spati al resolution determined by MTF curve. g) Schematic diagram of planar and non‐planar X‐ray imaging. h) Photographs of planar and non‐planar X‐ray images based on **Compound‐I**@paper.

As we know, soilless culture system is currently one of the fastest‐growing fields in new smart agriculture, which can greatly improve the utilization efficiency of agricultural resources such as space, water, and nutrition.^[^ ^46^ ^]^ However, plant roots in containers are more susceptible to environmental changes. In addition, plant roots are prone to lesions when they are exposed to direct solar radiation and excessive irrigation or insufficient irrigation. Therefore, real‐time monitoring of the healthy growth of plants is crucial. Unfortunately, traditional observation methods are often time‐consuming, labor‐intensive, destructive, and difficult to obtain comprehensive and accurate information. By contrast, the plant root X‐ray imaging system is a high‐tech device specifically designed for non‐destructive detection of plant root structure and functional status. It can scan plant roots in the container through high‐resolution X‐ray imaging technology and convert image data into analyzable information, timely understanding key parameters such as root morphology, length, diameter, and their changing trends over time. X‐ray scintillation plays an indispensable role in plant root X‐ray imaging system, and both organic Cu(I) iodides exhibit excellent scintillation performances due to their efficient broadband emission, negligible self‐absorption, and effective heavy atom effects. Figure S18 (Supporting Information) shows the X‐ray absorption coefficients of **Compound‐I** and **Compound‐II** at different X‐ray photon energies using the XCOM photon cross section database,^[^ ^47^ ^]^ which is comparable to Lu_3_Al_5_O_12_:Ce (LuAG:Ce), Bi_4_Ge_3_O_12_ (BGO), Cs_3_Cu_2_I_5_, and CsPbBr_3_. This result suggests that both organic Cu(I) iodides have outstanding stopping capacity. **Figure** **4**a shows the RL spectra of **Compound‐I** and **Compound‐II**, which exhibit similar spectral profile with the PL spectra. This result illustrates that RL and PL in organic Cu(I) iodides should originate from the same radiative recombination pathway. Using LuAG:Ce with a light yield of 25000 photons per MeV as a reference, the relative light yield of **Compound‐I** and **Compound‐II** was calculated to be 110200 and 40200 photons per MeV, respectively. Compared to **Compound‐II**, **Compound‐I** has a higher light yield, mainly due to its larger Stokes shift and lower non‐radiative decay rate, which is beneficial for light output.^[^ ^22^ ^]^ To best our knowledge, the light yield of **Compound‐I** represents the state‐of‐the‐art value reported so far. Figure S19 (Supporting Information) shows the RL spectra of **Compound‐I** and **Compound‐II** under different X‐ray dose rates, and the RL intensity increases linearly with the increase of X‐ray dose rate (8.9 mGy_air_/s‐92.5 mGy_air_/s). Moreover, the detection limits of **Compound‐I** and **Compound‐II** were determined to be 65 nGy_air_/s and 115 nGy_air_/s when the signal‐to‐noise ratio is equal to 3 (Figure 4b). Particularly, the ultra‐low detection limit of **Compound‐I** is nearly 84.6 times lower than the dose rate required for a standard medical X‐ray diagnosis (5.5 µGy_air_/s). Figure 4c shows the light yield versus detection limit for other reported lead‐free metal halide scintillators in the literatures. High light yield and low detection limit are two essential requirements for the next‐generation high‐performance X‐ray scintillator. Simultaneously realizing light yield > 110200 photons per MeV and detection limit < 70 nGy_air_/s is rarely reported in lead‐free metal halide scintillators. Figure 4d depicts the RL mechanism of organic Cu(I) iodides. Upon X‐ray irradiation, the heavy atoms in **Compound‐I** and **Compound‐II** can absorb the X‐ray and generate a large number of high‐energy hot electrons, and then generate secondary electron‐hole pairs via inelastic electron scattering and the Auger process.^[^ ^48^ ^]^ Subsequently, the generated electrons undergo thermalization and produce free carriers and excitons. Through rapid radiative recombination of thermally generated electron/hole pairs,^[^ ^49^ ^]^ a bright yellow RL emission can be observed.

Considering that **Compound‐I** exhibits superior X‐ray scintillation performance, we employed **Compound‐I**@paper film as the X‐ray scintillation screen for X‐ray imaging. Figure S20 (Supporting Information) shows the schematic diagram of plant root X‐ray imaging system. When the plant roots in nutrient solution are placed between the X‐ray source and the scintillation screen, the plant roots can be observed clearly (Figure 4e). In particular, the plant root X‐ray imaging system is a non‐destructive in‐situ analysis system that can comprehensively analyze all parts of the plant root. In addition, this system can conduct long‐term dynamic monitoring of plant root growth at different stages, which can effectively avoid damage to fragile parts such as plant root tips. The **Compound‐I**@paper scintillation screen can achieve a high X‐ray imaging spatial resolution of 21.6 lp mm^−1^ at a modulation transfer function (MTF) of 0.2 (Figure 4f). This value is superior to other scintillation screens prepared by mixing polymer matrix with lead‐free metal halides, such as (TMAA)_2_Cu_4_Br_6_/PMMA (15.6 lp mm^−1^),^[^ ^50^ ^]^ Cs_3_Cu_2_I_5_:0.1%Tl^+^/PDMS (10.2 lp mm^−1^),^[^ ^51^ ^]^ Cs_6_Cu_3_AgBr_10_/PDMS (8.06 lp mm^−1^),^[^ ^52^ ^]^ and C_38_H_36_P_2_SbCl_5_/PVDF (10.2 lp mm^−1^).^[^ ^53^ ^]^ The ultra‐high spatial resolution of **Compound‐I**@paper scintillation screen is due to the low scattering and absorption of X‐rays by the filter paper, as well as the uniform dispersion of **Compound‐I** microcrystals on the filter paper can effectively suppress optical crosstalk. Also noteworthy, **Compound‐I**@paper film exhibits remarkable irradiation stability, and the RL intensity shows negligible reduction under continuous X‐ray irradiation (0.19 mGy_air_/s) for 60 min (total dose: 684 mGy_air_, Figure S21, Supporting Information).

Subsequently, a comparative experiment of planar and non‐planar X‐ray imaging was constructed based on **Compound‐I**@paper film, respectively. In planar X‐ray imaging, an irregular object is placed at the front end of the film (Figure 4g, top panel). The result indicates that the distance between the irregular object and the scintillation screen causes random secondary photons to interfere with the effective optical signals, resulting in optical crosstalk and bringing about blurring and gradual halo issues. In non‐planar X‐ray imaging (Figure 4g, bottom panel), flexible scintillation screen can be tightly attached to irregular object, allowing clear visibility of the edges of the irregular object and effectively avoiding light scattering and crosstalk, thus achieving better imaging quality (Figure 4h).^[^ ^54^ ^]^ The above results fully demonstrate the significant advantages of **Compound‐I**@paper flexible scintillation screens in non‐planar X‐ray imaging.

**Figure 5:**
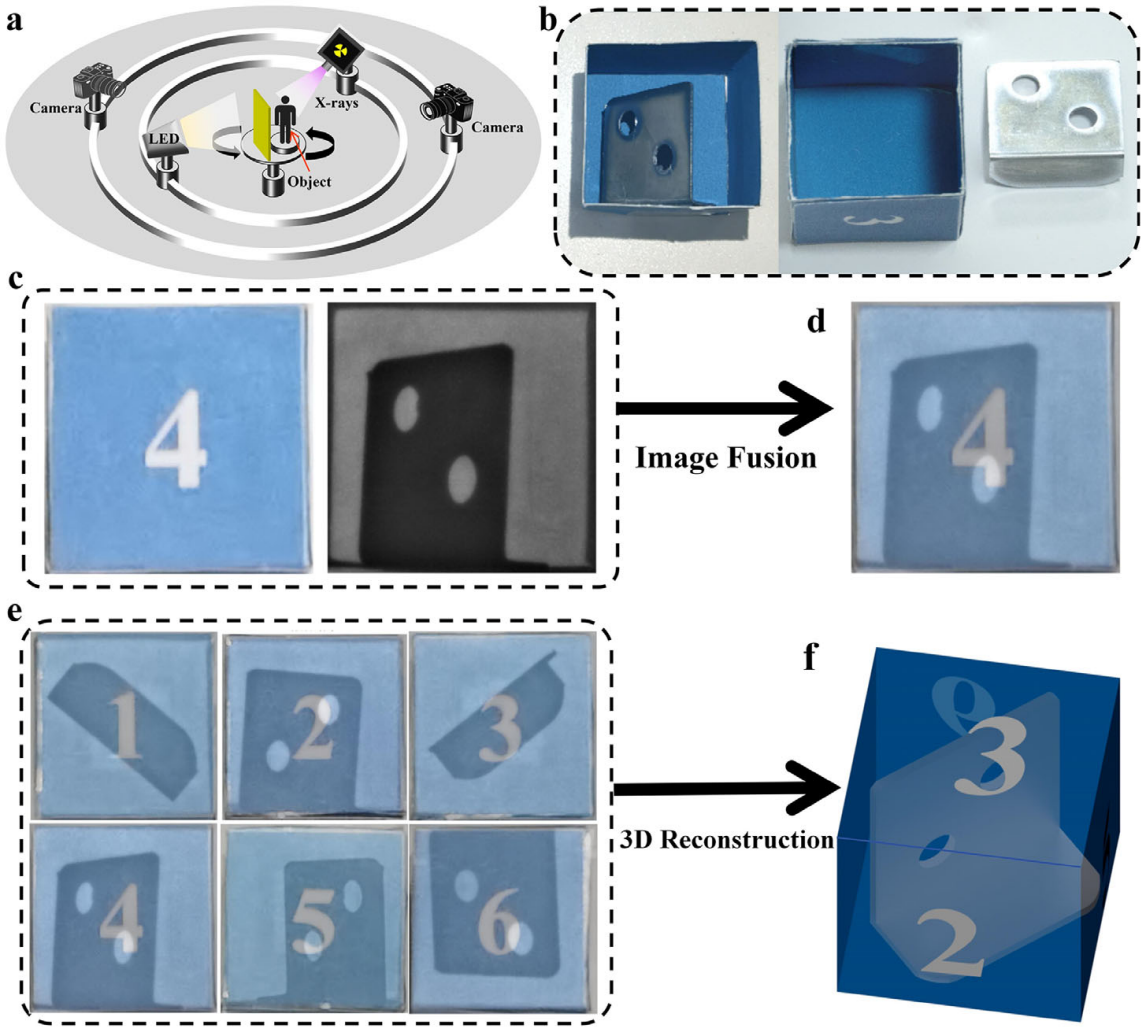
a) Scheme of the cooperative white light and X‐ray imaging. b) Photographs of an opaque cardboard box inside and outside. c) White light imaging and X‐ray imaging. d) White light and X‐ray image fusion. e) Fusion images under various angles. f) 3D image reconstruction.

Multispectral image fusion technology can integrate different types of spectra and obtain more comprehensive, reliable and rich feature data, so it has been widely used in medical imaging, remote sensing, food detection, and so forth.^[^ ^55^, ^56^ ^]^ Especially in the field of medical imaging, white light image emphasizes the appearance contour, while X‐ray image reflects the inorganic bone texture. The dual‐mode optical response of PL and RL in **Compound‐I**@paper film prompts us to explore its application in multispectral image fusion. In **Figure** **5**a, a new device for cooperative white light and X‐ray imaging is constructed based on the **Compound‐I**@paper film, which consists of a 460 nm LED array, an X‐ray source, a **Compound‐I**@paper film, a camera and a guide rail. Particularly, the light source and the camera can rotate under the support of the guide rail. In WLED mode, **Compound‐I**@paper is excited by 460 nm LED array and emits bright white emission, and the appearance contour of the target object can be obtained. Similarly, the X‐ray imaging can be achieved when the target object is irradiated by X‐ray, but the position of the camera is switched to the opposite side.

Subsequently, an experimental model was constructed to simulate the application of image fusion in the medical field, in which an L‐shaped iron sheet was fixed inside an opaque cardboard box, as shown in Figure 5b. Under WLED irradiation, the appearance contour of the cardboard box can be captured clearly (Figure 5c). Because L‐shaped iron sheet has a stronger X‐ray absorption capacity compared to cardboard box, the outline of L‐shaped iron sheet can be clearly seen under X‐ray irradiation. By fusing white light and X‐ray images, detailed information about the external shape and internal structure of the target object can be obtained (Figure 5d). Considering that X‐ray images contain more detailed information about objects inside the opaque cardboard box, the optimal multispectral fusion image can be obtained by increasing the weight coefficient of the X‐ray image (Figure S22, Supporting Information). Figure 5e exhibits the multiangle image fusion of the target object, and the images of the cardboard box and L‐shaped iron sheet under various angles were obtained, which allows for the realization of the 3D stereoscopic structure of the cardboard box was constructed through 3D reconstruction software of Maxon Cinema 4D (Figure 5f).

**Figure 6:**
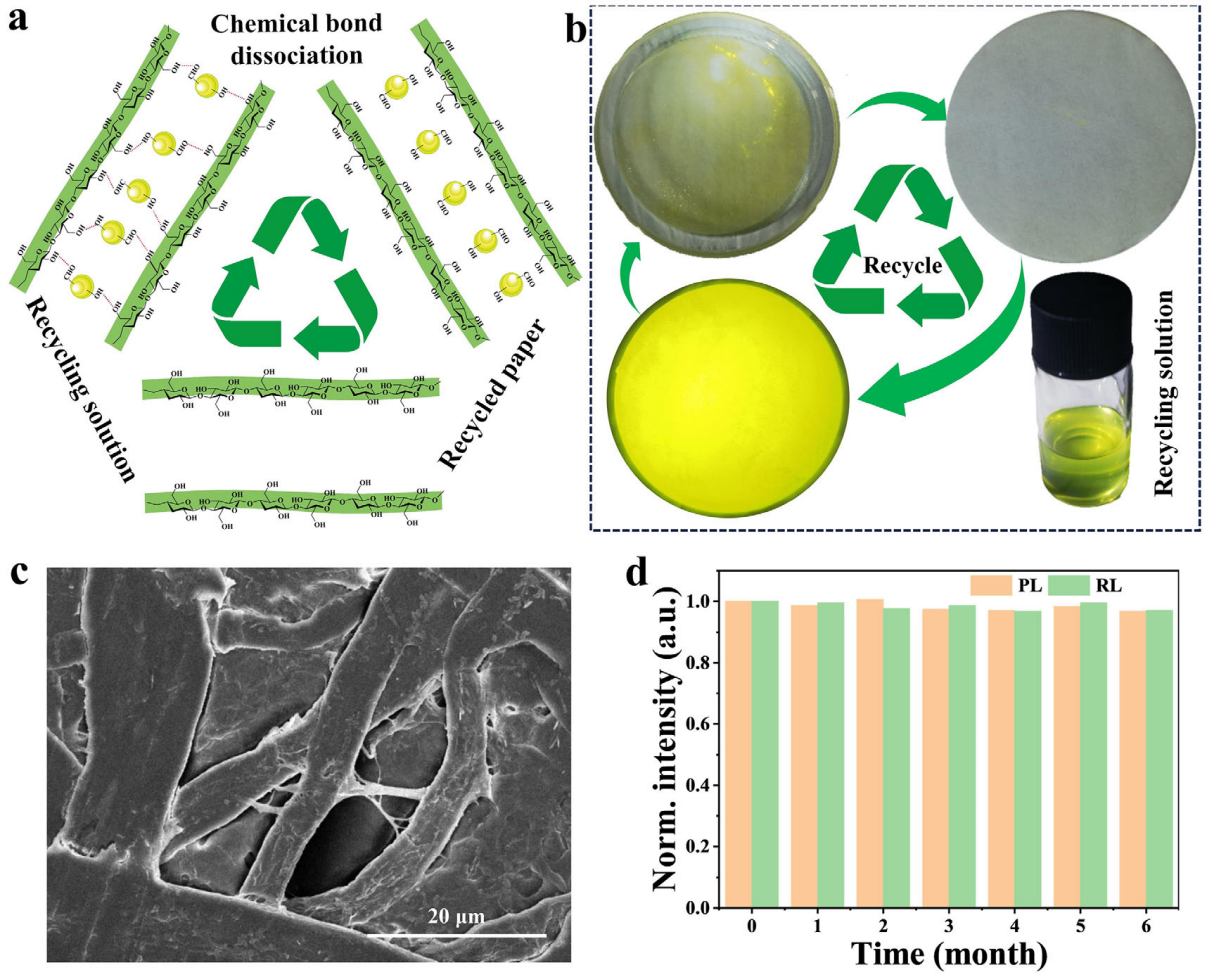
a) Scheme of **Compound‐I**@paper recovery and reprocessing. b) Images of the recovery system in daylight (recycling solution) and under 365 nm irradiation (filter paper). c) Top‐view SEM image of the recycled filter paper. d) Stability of the normalized PL and RL intensity of the recycled **Compound‐I**@paper film.

Generally, the emission layer of WLED as well as X‐ray scintillation screen are mainly prepared by mixing luminescent materials and polymers. The primary source of polymers is non‐renewable petroleum resources, and the non‐recyclability of these materials increases the challenge of achieving carbon neutrality. Consequently, recycling and reusing biological resources can play a pivotal role in reducing carbon footprints and facilitating the transition from petroleum‐based materials to renewable resources.^[^ ^43^ ^]^ Combined with the excellent solubility of **Compound‐I** in DMF solution, we propose a mechanism for **Compound‐I**@paper recovery and reprocessing, as shown in **Figure** **6**a. With the assistance of ultrasound treatment, the chemical bond between **Compound‐I** and cellulose is preemptively dissociated. Subsequently, **Compound‐I** microcrystals were rapidly dissolved in DMF solution, and **Compound‐I** solution and filter paper frame can be obtained, respectively. Particularly, the reprocessed **Compound‐I**@paper film can be obtained again by adding an appropriate amount of **Compound‐I** precursor to the recovery solution and using the “ancient fabric dyeing process”. Figure 6b shows the proof‐of‐concept experiment for the recovery of **Compound‐I**@paper film, which retains the filter paper with a perfect framework structure and the recovery solution. In addition, the top‐view SEM image of recovered **Compound‐I**@paper film is characterized (Figure 6c), and the result shows that the structure of cellulose is well preserved, and **Compound‐I** microcrystals are completely dissolved in DMF solution. In addition, the PL and RL characteristics of **Compound‐I**@paper film can be well preserved after the recycling process, thus making it reusable. As proof, **Compound‐I**@paper film was prepared again using the same preparation process, and it showed perfect reducibility in both PL and RL responses (Figure 6d). Against the backdrop of fossil fuel shortages and worsening global warming, this material is almost harmless to the environment. Moreovcer, **Compound‐I**@paper film exhibits excellent biodegradability and recyclability within the natural soil environment. To demonstrate this, when the **Compound‐I**@paper film is buried in the soil, it can be effectively decomposed by microorganisms in nature within 20 days and transformed into absorbable nutrients (Figure S23, Supporting Information), which are included in the carbon cycle. Therefore, the efficient PL and RL dual‐mode response of this green and recyclable paper‐based customizable large‐area flexible film makes it show great application potential in smart agriculture.

## 3 Conclusion

In summary, two new 0D organic Cu(I) iodides of **Compound‐I** and **Compound‐II** were synthesized through supramolecular self‐assembly. Under blue light excitation, both emit broadband yellow emission with near‐unity PLQY, which stem from the radiation transition of TSCT and CC excited state. Interestingly, **Compound‐II** shows the reversible PL switching between yellow emission and non‐emission under heat or methanol treatment, accompanied by a reversible crystal structure conversion between **Compound‐II** and **Compound‐III**. Moreover, **Compound‐I** and **Compound‐II** exhibit significant X‐ray response, and **Compound‐I** has an ultra‐high light yield of 110200 photons per MeV and an ultra‐low detection limit of 65 nGy_air_/s. Afterwards, **Compound‐I** microcrystals were uniformly precipitated in situ on cellulose of the filter paper, thus successfully preparing customizable large‐area flexible **Compound‐I**@paper film. Using **Compound‐I** as the emission layer of single‐component WLED and X‐ray scintillation screen, which exhibit a luminous efficiency of 114.5 lm W^−1^ and a spatial resolution of 21.6 lp mm^−1^, respectively, demonstrating great application potential in plant growth lighting and plant root X‐ray imaging. Combined multispectral image fusion and multiangle imaging, the 3D stereoscopic structure of L‐shaped iron sheet in the opaque cardboard box was reconstructed. In addition, the end‐of‐life **Compound‐I**@paper film can be recycled in DMF solution, which exhibits remarkable recycling characteristic. Considering that filter paper can be biodegraded in natural soil environments, providing nutrients for plant growth and participating in carbon cycling. Therefore, this customizable large‐area flexible luminescent film has comprehensive advantages such as simple preparation, recyclability, biodegradability, and sustainability, which can establish a bifunctional platform involving plant growth lighting and plant root X‐ray imaging, providing a promising method for the sustainable development of smart agriculture.

## 4 Experimental Section

### Materials

Cuprous iodide (CuI, 99%) was purchased from Sigma‐Aldrich reagent. Calcium iodide (CaI_2_, 99%), 15‐crown‐5 (C_10_H_20_O_5_, 99%), and 18‐crown‐6 (C_12_H_24_O_6_, 99%) were purchased from Macklin reagent. Methanol (CH_3_OH, 99.5%), acetone (C_3_H_6_O, 99.5%), and N, N‐dimethylformamide (DMF, 99.5%) were purchased from Aladdin reagent. All chemicals were used directly as received.

### Synthesis of Bulk Crystals of Compound‐I

4 mmol CuI, 1 mmol CaI_2_, and 2 mmol 15‐crown‐5 were dissolved in DMF (5 mL) to form a transparent solution. Subsequently, the above solution is slowly evaporated at 40°C, and the bulk crystals of **Compound‐I** can be obtained.

### Synthesis of Bulk Crystal of Compound‐II

4 mmol CuI, 1 mmol CaI_2_, and 2 mmol 18‐crown‐6 were dissolved in the mixed solvents of acetone (8 mL) and methanol (3 mL) to form a transparent solution. Subsequently, the above solution is slowly evaporated at room temperature, and the bulk crystals of **Compound‐II** can be obtained.

### Synthesis of Bulk Crystal of Compound‐III

For synthesizing **Compound‐III**, only acetone (8 mL) was used as the solvent under otherwise identical conditions as **Compound‐II**.

### Fabrication of Compound‐I@paper Flexible Luminescent Film

8 mmol CuI, 2 mmol CaI_2_, and 4 mmol 15‐crown‐5 were dissolved in DMF (20 mL) to form a transparent solution. Then, the filter paper is completely immersed in the solution, and the precursor is fully diffused into the filter paper under ultrasonic treatment. After removing the filter paper from the precursor and placing it onto a hot plate at 40 °C, a **Compound‐I**@paper flexible luminescent film can be obtained within 350 s, and the more detailed processes are shown in Figure 3a.

### Recycling Process of Compound‐I@paper Flexible Luminescent Film

The **Compound‐I**@paper flexible luminescent film was immersed in a 20 ml DMF solution and supplemented with ultrasonication, the recovered filter paper and the DMF solution of **Compound‐I** can be obtained. The recovered filter paper was dried, and an appropriate amount of precursor was added to the recovered solution to ensure that the concentration of the recovered solution remains consistent with that when the film was first prepared. The dried filter paper was then fully immersed in the solution to prepare a reusable **Compound‐I**@paper flexible luminescent film.

### Characterization

Crystal structures were performed by X‐ray single crystal diffractometer (SCXRD, XtaLAB Synergy Custom). Powder X‐ray diffraction (PXRD) patterns were recorded using a Bruker D8 Advance diffractometer with Cu Kα radiation in the 2θ range of 4‐40°. Scanning electron microscopy (SEM) and energy dispersive X‐ray spectroscopy (EDS) analyses were conducted using a Carl Zeiss Merlin high‐resolution scanning electron microscope equipped with an Oxford EDX detector. X‐ray photoelectron spectroscopy (XPS) was performed using Thermo Fisher Nexsa X instrument. Excitation and emission spectra were recorded using a Horiba Fluoromax‐4 spectrometer. Photoluminescence quantum yields (PLQYs) and temperature‐dependent emission spectra were measured using an Edinburgh FLS 1000 fluorescence spectrometer. Radioluminescence (RL) spectra and light yield were measured using an Edinburgh FL5 fluorescence spectrometer equipped with an X‐ray tube (Tungsten target, Moxtek). A commercial LuAG:Ce crystal was used as a reference sample to calculate the light yields of the samples. X‐ray imaging was collected by a homemade X‐ray imaging system equipped COMSOL camera under X‐ray tube excitation, and the object is about 10 cm away from the X‐ray source. The modulation transfer function (MTF) is an index to evaluate the spatial resolution of imaging system, which is defined as the transfer capability of the input signal modulation of spatial frequency. Using slanted‐edge method to obtain the MTF curve, we took the X‐ray images of a sharp edge from a piece of tungsten with the thickness of 1 mm.

### WLED Fabrication

The single‐component WLED was fabricated by coating **Compound‐I**@paper film on a 460 nm LED array, as shown in Figure S15b (Supporting Information). The emission spectra, CIE color coordinates, color rendering index, and correlated color temperature of the WLED were collected using the Hopoo HPCS6500 instrument.

## Conflict of Interest

The authors declare no conflict of interest.

## Supporting information



Supporting Information

Supporting Information

## Data Availability

The data that support the findings of this study are available from the corresponding author upon reasonable request.
