# Supplementary material for: Supramolecular Self‐Assembly of Organic Cu(I) Iodides as Green and Recyclable Paper‐Based Customizable Large‐Area Flexible Film for Plant Grow Lighting and Plant Root X‐Ray Imaging
Source: Adv Sci (Weinh). 2025 Oct 5;12(48):e13081. doi: 10.1002/advs.202513081 (PMC12752624; doi:10.1002/advs.202513081)
Supplement: Supplementary file 1 — Supporting Information [file ADVS-12-e13081-s002.docx]

**Supramolecular [Self-Assembly](https://webofscience.clarivate.cn/wos/alldb/full-record/WOS:001156430300001) of Organic Cu(I)** **Iodides** **as** **Green and Recyclable** **Paper-Based** **Customizable Large-Area Flexible Film for Plant Grow Lighting and Plant Root X-Ray Imaging**

Shengji Yuan,^†^ Hui Peng^†*^, Hairui Zhang,^†^ Ou Xu,^†^ Fei Wang,^†^ Wenchao Yang,^†^ Zhentao Du,^†^ and Bingsuo Zou^†*^

^†^Guangxi Key Laboratory of Processing for Non-ferrous Metals and Featured Materials, and School of Resources, Environment and Materials, Guangxi University, Nanning 530004, China.

E-mail: [penghuimaterial@163.com](mailto:penghuimaterial@163.com); zoubs@gxu.edu.cn

**Table S1.** Detailed crystallographic data for [Ca(15-crown-5)_2_]Cu_4_I_6_·2C_3_H_7_NO·H_2_O (**Compound-I**), [Ca_2_(18-crown-6)_4_]Cu_4_I_8_·6H_2_O (**Compound-II**), and [Ca(18-crown-6)]Cu_5_I_7_·3H_2_O (**Compound-III**).

| Empirical formula | **Compound-I** | **Compound-II** | **Compound-III** |
| --- | --- | --- | --- |
| Formula weight | 1660.36 | 2514.86 | 3322.70 |
| Temperature(K) | 273.00 | 297.00 | 301.00 |
| Crystal system | monoclinic | triclinic | monoclinic |
| Space group | P2_1_/c | P-1 | Pbmc |
| a (Å) | 10.5377(4) | 10.5148(5) | 10.648(2) |
| b (Å) | 16.2326(5) | 11.8832(5) | 14.444(5) |
| c (Å) | 29.1262(11) | 18.0885(11) | 22.674(6) |
| α (deg) | 90 | 106.065(2) | 90 |
| β (deg) | 92.615(1) | 102.301(2) | 90 |
| γ (deg) | 90 | 98.272(4) | 90 |
| Volume(Å^3^) | 4977.0(3) | 2071.91(19) | 3487.3(17) |
| Z | 4 | 1 | 2 |
| Density (calculated)  (g· cm^-3^) | 2.216 | 2.016 | 3.164 |
| Absorption coefficient  (mm^‑1^) | 5.560 | 4.186 | 10.563 |
| Data/restraints/parameters | 12331/349/541 | 10208/1/420 | 12208/1/455 |
| Goodness-of-fit on F^2^ | 1.032 | 1.035 | 1.033 |
| Final R indexes [I>=2σ (I)] | R_1_ = 0.0622  wR_2_ = 0.1449 | R_1_ = 0.0409  wR_2_ = 0.0863 | R_1_ = 0.0457  wR_2_ = 0.0163 |
| Final R indexes [all data] | R_1_ = 0.1293,  wR_2_ = 0.1815 | R_1_ = 0.0591  wR_2_ = 0.0944 | R_1_ =0.0387  wR_2_ = 0.0687 |

**Table S2.** Comparison of element concentrations obtained from EDS analysis of **Compound-I**, **Compound-II**, and **Compound-III**.

| Element | **Compound-I** | **Compound-II** | **Compound-III** |
| --- | --- | --- | --- |
| Cu (%) | 36.21 | 28.59 | 38.35 |
| Ca (%) | 9.04 | 14.20 | 7.66 |
| I (%) | 54.75 | 57.21 | 53.99 |

**Table S3.** Room temperature photophysical properties of **Compound-I** and **Compound-II**.

| Materials | PLE (nm) | PL (nm) | PLQY (%) | τ_av_ (μs) | k_r_ (s^-1^) ×10^4^ | k_nr_ (s^-1^) × 10^4^ |
| --- | --- | --- | --- | --- | --- | --- |
| **Compound-I** | 450 | 540 | 99.0 | 2.01 | 49.25 | 0.49 |
| **Compound-II** | 445 | 528 | 90.2 | 1.81 | 49.83 | 5.41 |

**Table S4.** Summary of the optical properties of metal halides and corresponding WLEDs reported in recent years.

| Materials | PLQY  (%) | PLE Range  (nm) | CIE | CRI | CCT  (K) | Luminous efficiency  (lm/W) | Ref. |
| --- | --- | --- | --- | --- | --- | --- | --- |
| DPCu_4_I_6_ | 93.5 | 240-450 | (0.36, 0.35) | 85.1 | 4415 | 18.4 | [1] |
| (Ph_3_MeP)_2_Cu_4_I_6_ | 69.0 | 400-500 | (0.41, 0.39) | 88 | 3500 | 65 | [2] |
| (TPP)_2_Cu_4_I_6_·2DMSO | 96.7 | 250-450 | (0.26, 0.30) | -- | 12449 | 50 | [3] |
| Mn-doped K_3_SbCl_6_ | 22.3 | 275-380 | (0.32, 0.30) | 82 | 4779 | 28.2 | [4] |
| Na_4_(18-crown-6)_5_In_2_Cu_4_ Br_14_·8H_2_O | 97 | 300-400 | (0.37, 0.42) | 75 | 4429 | 19.8 | [5] |
| CsPbBr_3_@silica-aerogel | 85.7 | - | (0.34, 0.34) | 90.5 | 5200 | 25.9 | [6] |
| (C_19_H_18_P)_2_Cu_4_I_6_ | 87.4 | 300-500 | (0.33, 0.33) | 86.7 | 5233 | 90.0 | [7] |
| Cs_3_Cu_2-2x_I_5_:2*x*Mn^2+^ | -- | 350-425 | (0.30, 0.36) | 77.9 | 6828 | 14.1 | [8] |
| [TMPDA]Cu_2_I_6_ | 26.7 | 250-330 | (0.33, 0.34) | 95 | 5552 | 30.5 | [9] |
| (TETA)InCl_6_·Cl·H_2_O:Sb^3+^ | 73.46 | 230-400 | (0.33, 0.33) | 90.5 | 5415 | 43.72 | [10] |
| Cs_4_PbBr_6_/CsPbBr_3_ | 55.41 | 300-500 | (0.34, 0.31) | - | 5458 | 64.56 | [11] |
| **Compound-I**@Paper | 99.0 | 240-500 | (0.31, 0.34) | 86.4 | 6439 | 114.5 | This work |

**Table S5.** Comparison with other LEDs currently reported for plant lighting.

| LED Species | CRI | CCT | Integration | Degradability | Recyclability | Ref. |
| --- | --- | --- | --- | --- | --- | --- |
| DPCu_4_I_6_ | 85.1 | 4415 | No | No | No | [1] |
| (Ph_3_MeP)_2_Cu_4_I_6_ | 88 | 3500 | No | No | No | [2] |
| (TPP)_2_Cu_4_I_6_·2DMSO | -- | 12449 | No | No | No | [3] |
| Mn-doped K_3_SbCl_6_ | 82 | 4779 | No | No | No | [4] |
| Na_4_(18-crown-6)_5_In_2_Cu_4_ Br_14_·8H_2_O | 75 | 4429 | No | No | No | [5] |
| CsPbBr_3_@silica-aerogel | 90.5 | 5200 | No | No | No | [6] |
| (C_19_H_18_P)_2_Cu_4_I_6_ | 86.7 | 5233 | No | No | No | [7] |
| Cs_3_Cu_2-2x_I_5_:2xMn^2+^ | 77.9 | 6828 | No | No | No | [8] |
| [TMPDA]Cu_2_I_6_ | 95 | 5552 | No | No | No | [9] |
| (TETA)InCl_6_·Cl·H_2_O:Sb^3+^ | 90.5 | 5415 | No | No | No | [10] |
| Cs_4_PbBr_6_/CsPbBr_3_ | - | 5458 | No | No | No | [11] |
| **Compound-I**@Paper | 86.4 | 6439 | Yes | Yes | Yes | This work |

Table S6. Light yield and detection limit of recently reported lead-free metal halide scintillators.

| Materials | Light yield (Photons/MeV) | Detection limit (nG_airy_/s) | Ref. |
| --- | --- | --- | --- |
| Rb_2_CuBr_3_ | 91056 | 121.5 | [12] |
| (MTPP)_2_Cu_4_I_6_ | 63700 | 124.4 | [13] |
| [AEPipz]CuBr_3_·Br·H_2_O | 62400 | 95.7 | [14] |
| Cs_3_Cu_2_I_5_: Tl@PMMA | 48800 | 305 | [15] |
| [BAPMA]Cu_2_Br_5_ | 43744 | 74 | [16] |
| TPA_2_Cu_2_I_4_@PVDF | 40124 | 126 | [17] |
| (Cu_2_I_2_(3,4-DMP)_4_ | 38031 | 106.7 | [18] |
| CuI(Cl-py) | 22000 | 250 | [19] |
| (DIET)_3_Cu_3_Br_3_ | 20000 | 189 | [20] |
| (Bmpip)_2_Cu_2_Br_4_ | 16000 | 710 | [21] |
| C_6_H_8_N_2_OCuI | 52000 | 43.14 | [22] |
| [C_20_H_20_P]_2_[MnBr_4_] | 10700 | 185.9 | [23] |
| Cs_4_Cd_0.75_Mn_0.25_Bi_2_Cl_12_@PDMS | 34450 | 183.6 | [24] |
| (PPN)_2_SbCl_5_ | 49000 | 191.4 | [25] |
| [TPPen]_2_Mn_0.9_Zn_0.1_Br_4_ | 68000 | 204.1 | [26] |
| Cs_2_TeCl_6_ | 38523 | 258 | [27] |
| Cu_4_I_4_(DPPPy)_2_ | 29000 | 88.2 | [28] |
| **Compound-II** | 40200 | 115 | This work |
| **Compound-I** | 110200 | 65 | This work |


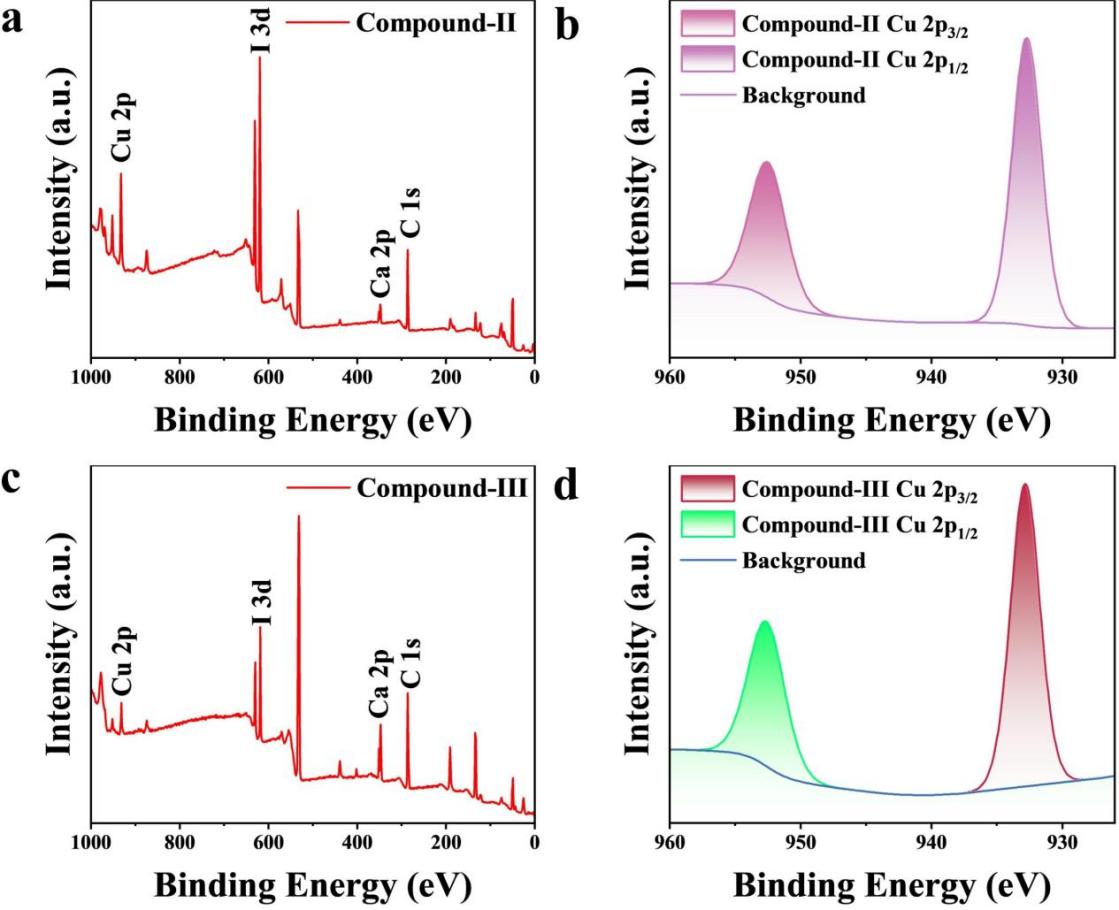


**Figure S1.** (a) XPS spectrum of **Compound-II**. (b) Fine XPS spectrum of Cu 2p for **Compound-II**. (c) XPS spectrum of **Compound-III**. (d) Fine XPS spectrum of Cu 2p for **Compound-III**.

**
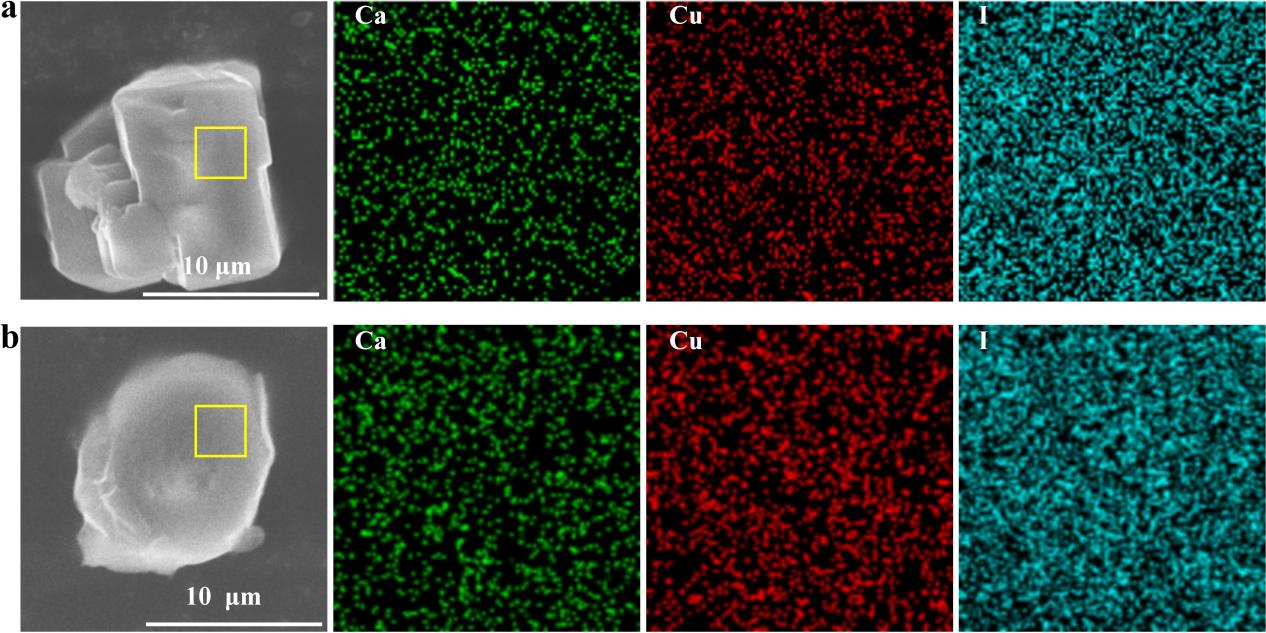
**

**Figure S2.** (a) SEM image of **Compound-II** and the corresponding element mapping images of Ca, Cu, and I, respectively. (b) SEM image of **Compound-III** and the corresponding element mapping images of Ca, Cu, and I, respectively.


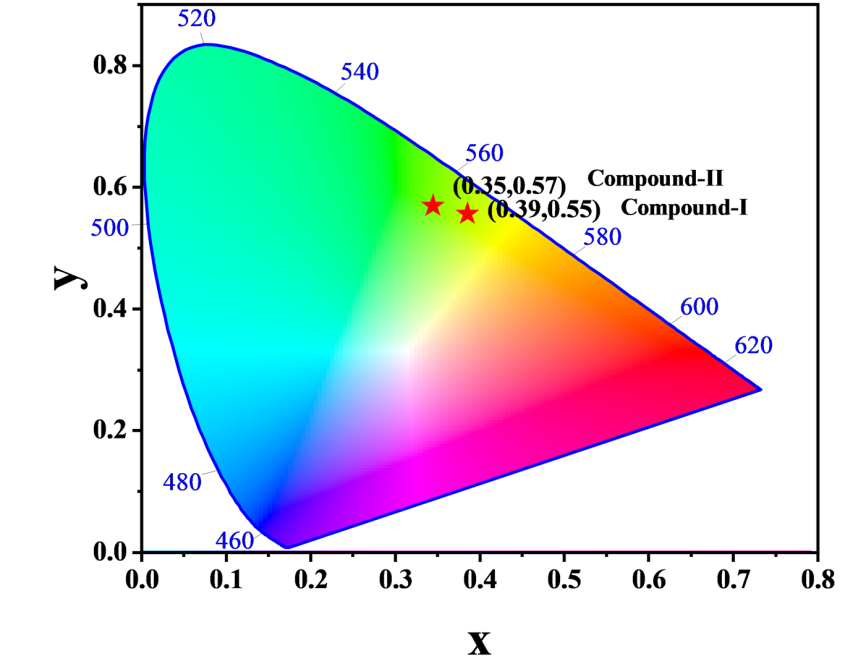


**Figure S3.** CIE color coordinates of **Compound-I** and **Compound-II**.


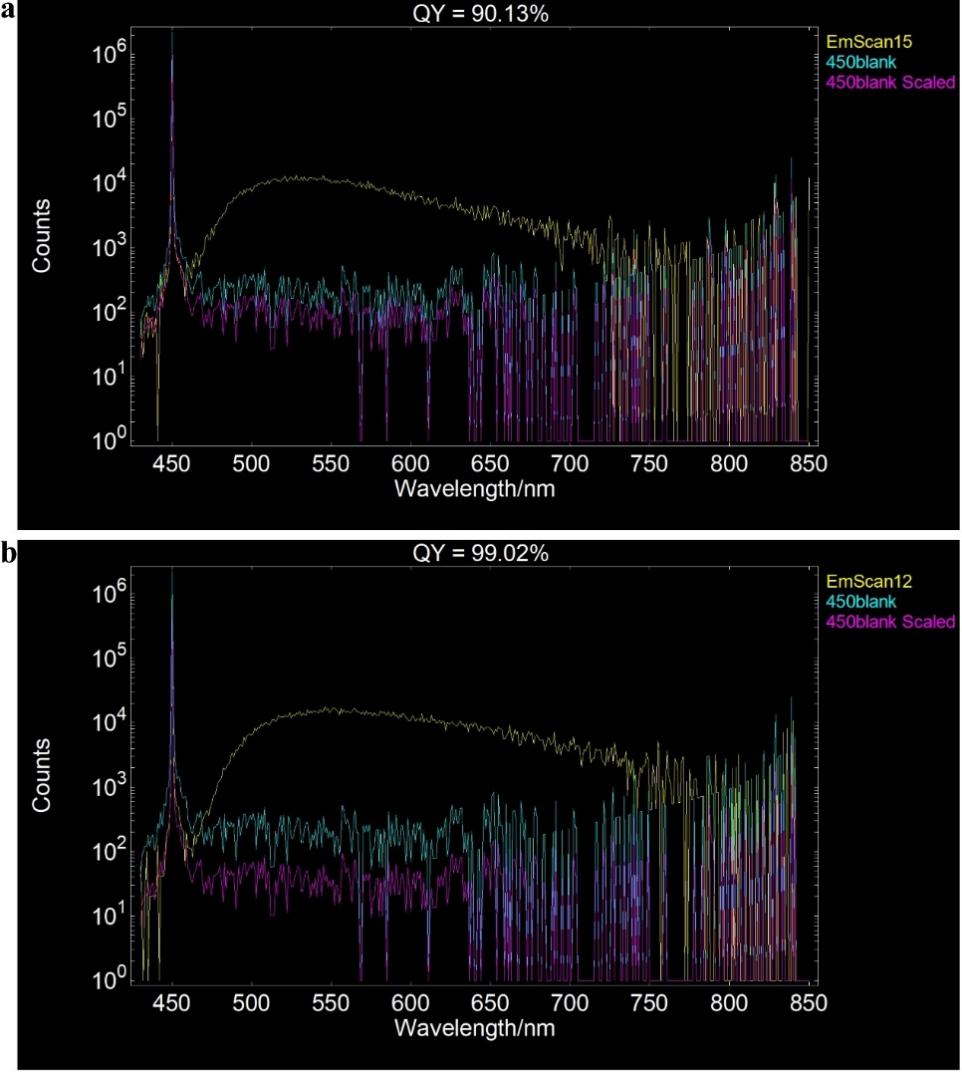


**Figure S4.** PLQYs of (a) **Compound-I** and (b) **Compound-II**.


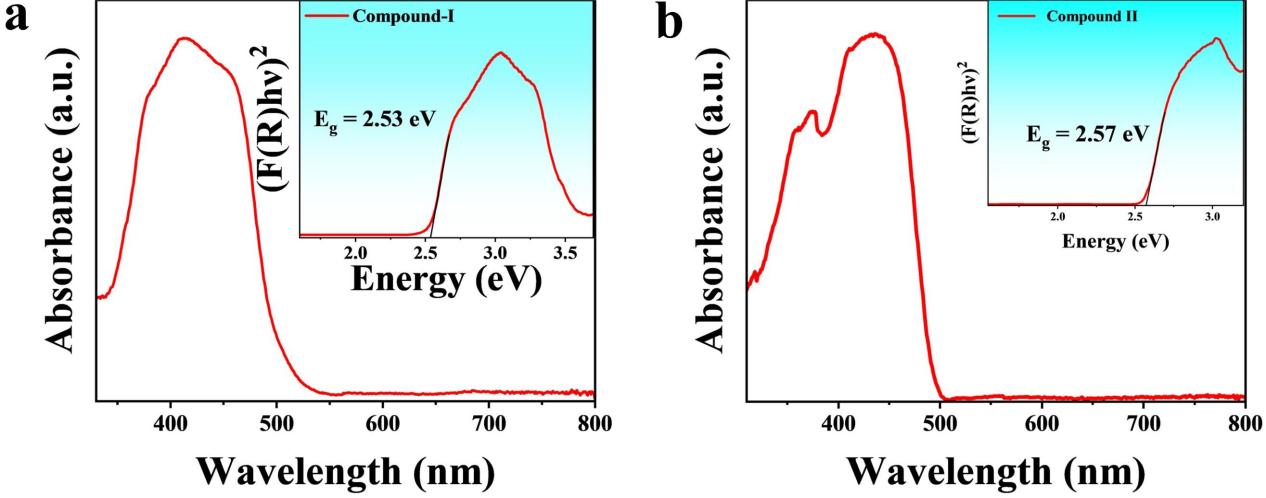


**Figure S5.** Absorption spectra of (a) **Compound-I** and (b) **Compound-II**.


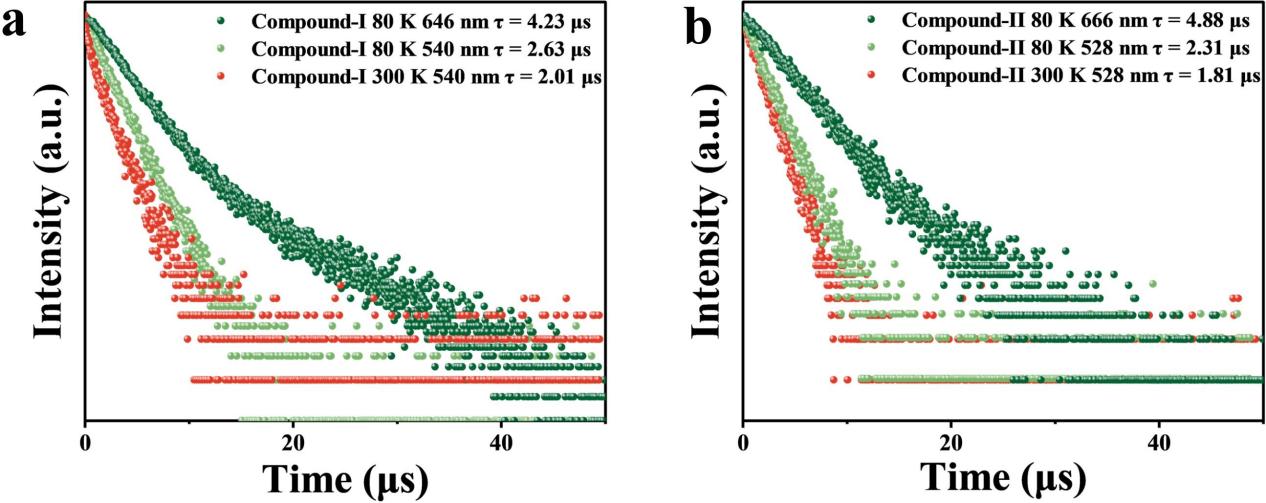


**Figure S6.** Time-resolved PL spectra of (a) **Compound-I** and (b) **Compound-II** at 300 and 80 K, respectively.


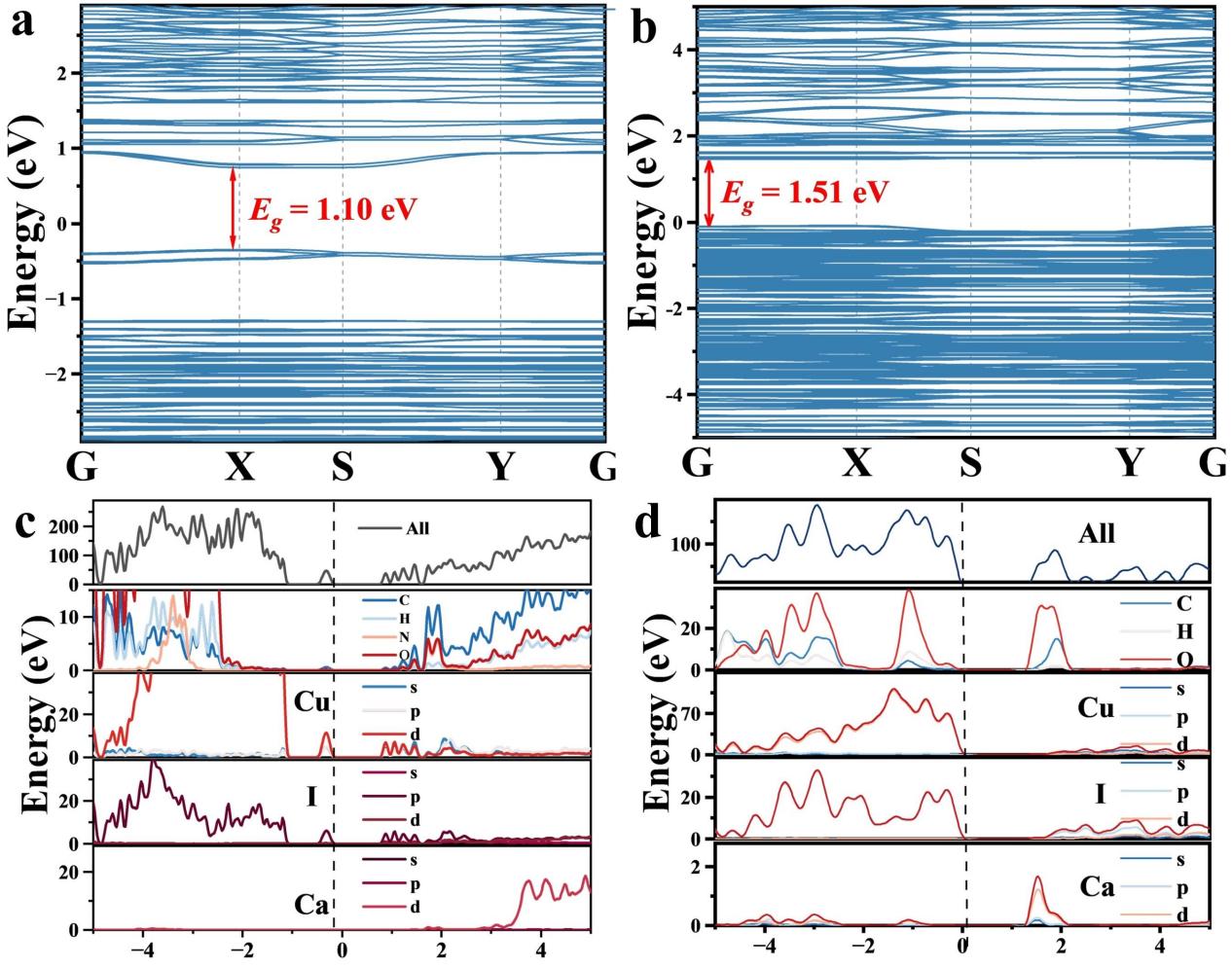


**Figure S7.** Band structure and DOS of (a, c) **Compound-I** and (b, d) **Compound-II**.

**Note:** Density-functional theory (DFT) calculations based on crystal cell structure were performed using the projection-augmented wave (PAW) method as implemented in the Vienna Ab initio Simulation Package (VASP) code.^[29]^ The plane-wave cutoff energy was set to 400 eV. The generalized gradient approximation (GGA) Perdew-Burke-Ernzerhof (PBE) was employed as the exchange-correlation functional.^[30]^ For structural geometry optimization, the energy convergence and residual forces on each atom are set as 10^-6^ and 0.02 eV/Å, respectively.


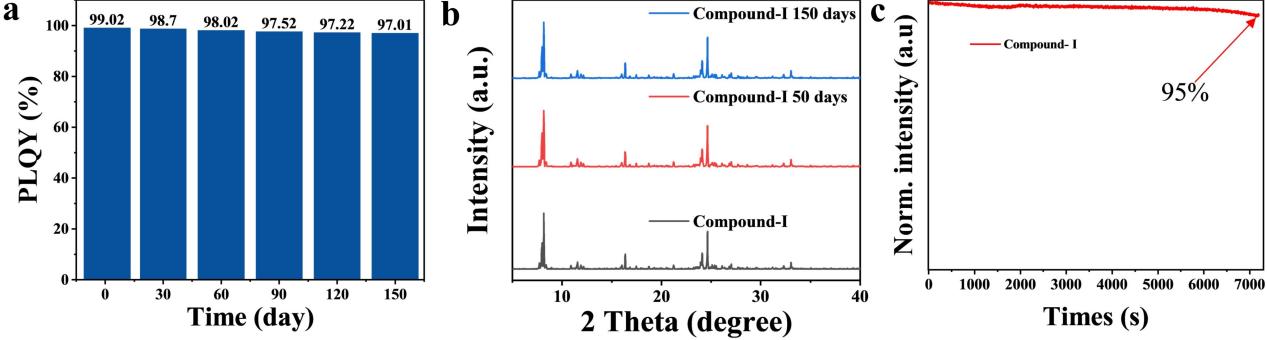


**Figure S8.** PLQY of **Compound-I** under different storage duration (relative humidity = 55%). (b) XRD patterns of **Compound-I** stored in air for 150 days. (c) PL intensity of **Compound-I** upon 450 nm continuous irradiation for 7200 s.

**
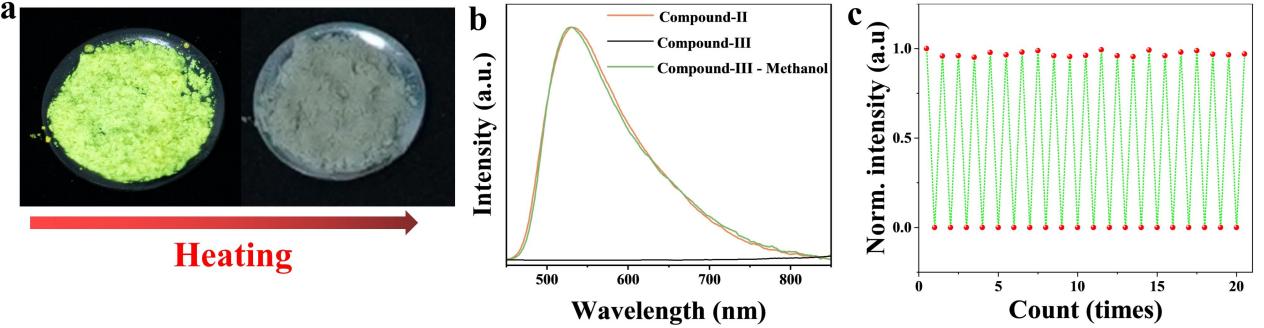
Figure S9.**  (a) Optical images of reversible PL conversion of **Compound-II** after heat treatment. (b) PL spectra of **Compound-III**, after methanol treatment of **Compound-III**, and **Compound-II**. (c) **Compound-II** after heat and methanol treatment.


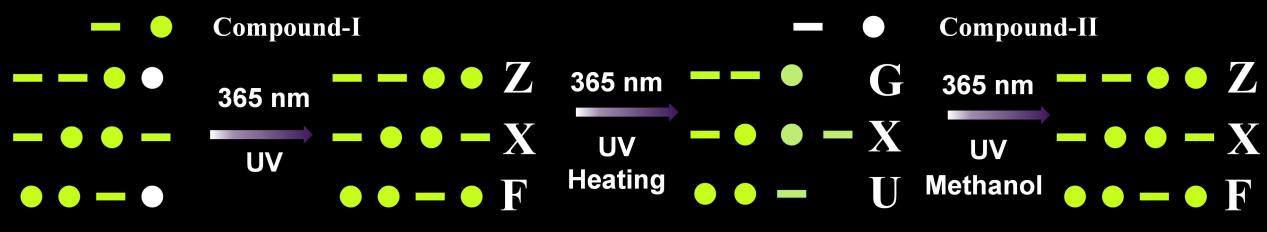


**Figure S10.** Morse code information delivery system based on organic Cu(I) iodides.

**Note:** Confidential numerical information is encrypted using Morse code, with **Compound-I** and **Compound-II** being placed into the corresponding array points. Under 365 nm UV light irradiation, the array points containing **Compound-I** and **Compound-II** emit yellow light, displaying the incorrect password “ZXF”. Upon heat treatment, **Compound-II** is converted to **Compound-I****II**, resulting in fluorescence quenching. At this stage, the correct information “GXU” becomes readable. After treatment by methanol, the decrypted information can be hidden, during which **Compound-III** is reverted to **Compound-II** to emit yellow light, thereby concealing the correct password “GXU” with “ZXF” once again and re-encrypting the information.

**
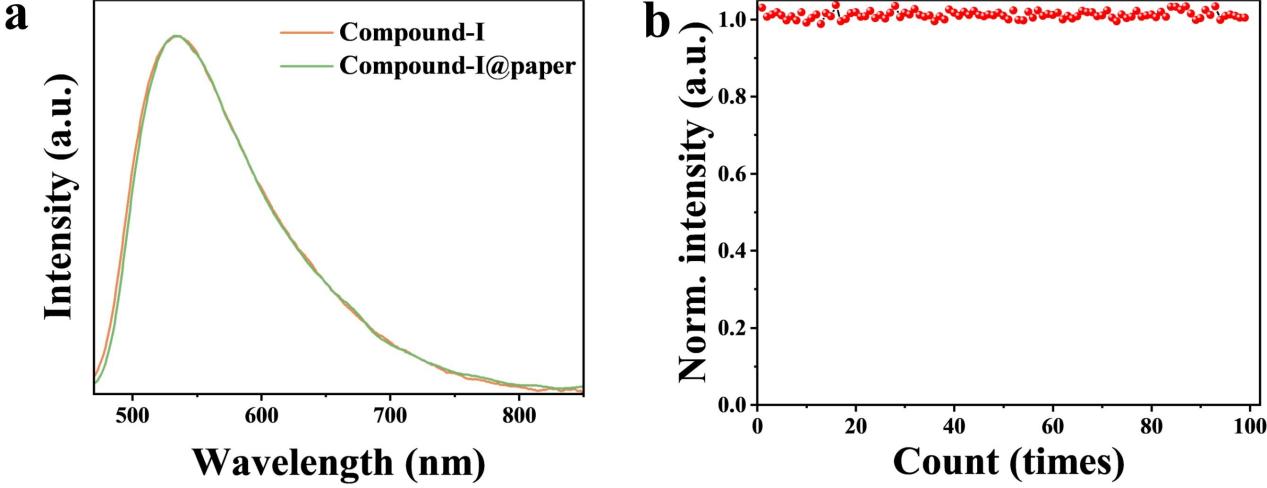
**

**Figure S11.**  (a) PL spectra of **Compound-I** and **Compound-I**@paper film. (b) Emission intensity of **Compound-I**@paper after 100 consecutive folds.


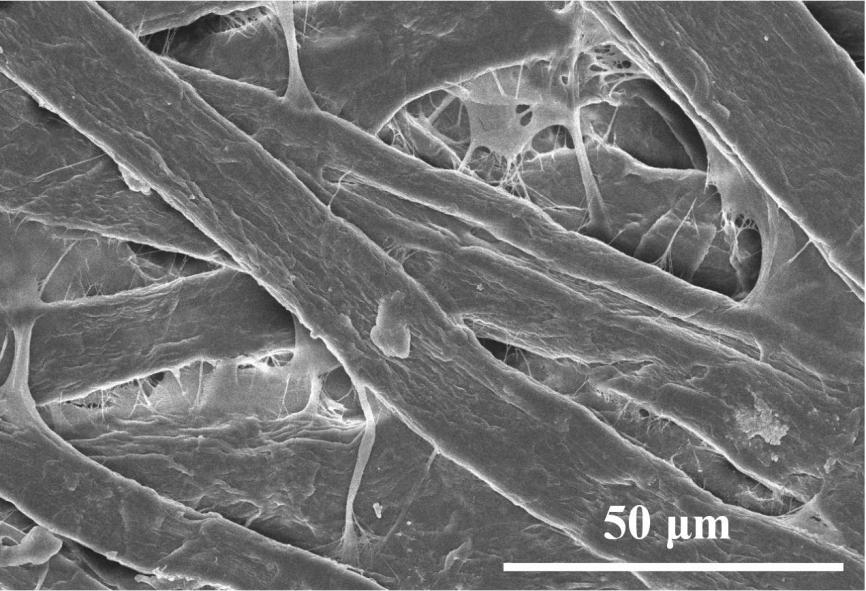


**Figure S12.** Top-view SEM image of the filter paper.


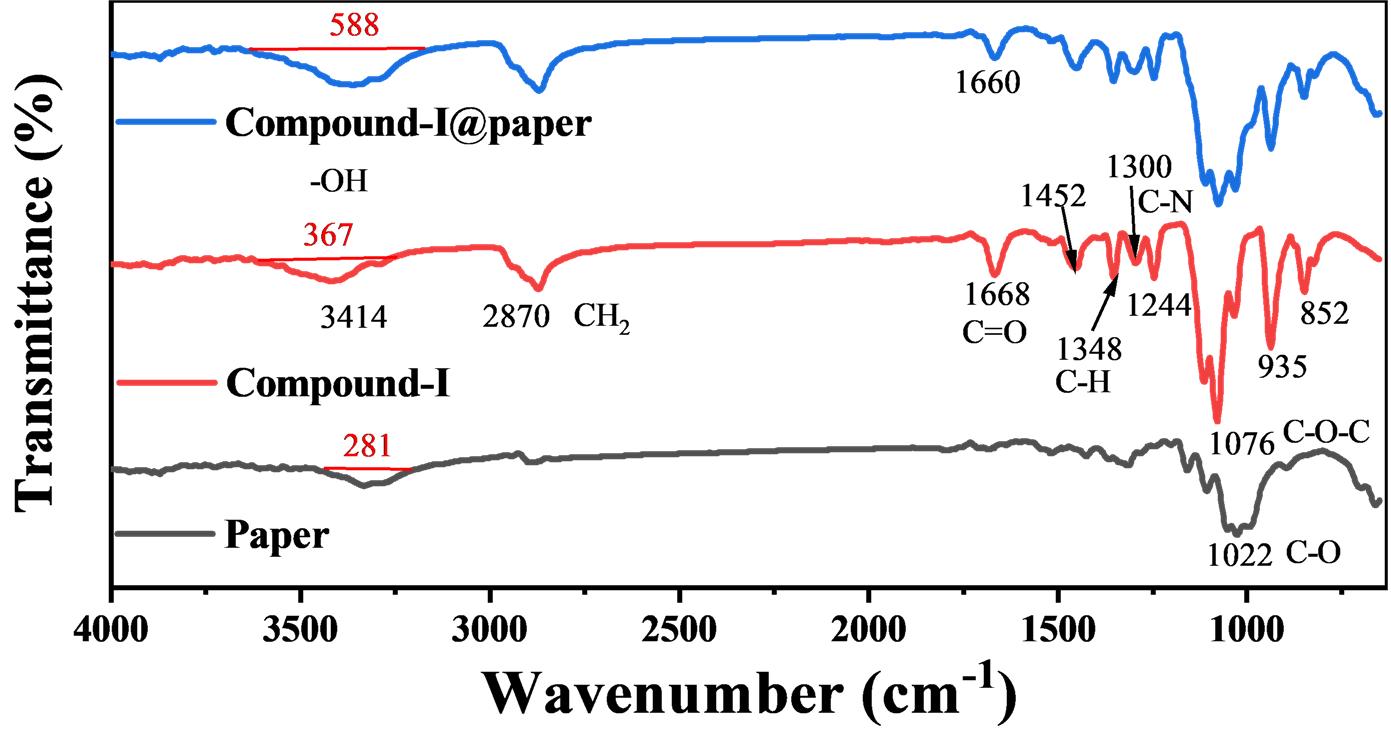


**Figure S13.** Fourier-transform infrared spectroscopy (FTIR) spectra of paper, **Compound-I**, and **Compound-I**@paper, respectively.

**Note:** FTIR spectra show that the peak width of -OH at about 3414 cm^-1^ increases from 367 cm^-1^ for **Compound-I** to 588 cm^-1^ for **Compound-I**@paper, which is due to the formation of a more stable hydrogen bond between **Compound-I** and cellulose. Compared to **Compound-I**, when the **Compound-I** microcrystals were uniformly precipitated in situ on cellulose of filter paper, the vibrational peaks of C=O in DMF shifts from 1668 cm^-1^ to 1660 cm^-1^, which should be caused by the reaction of -CHO with -OH. There strong intermolecular interactions are beneficial for **Compound-I** to firmly bind to cellulose.


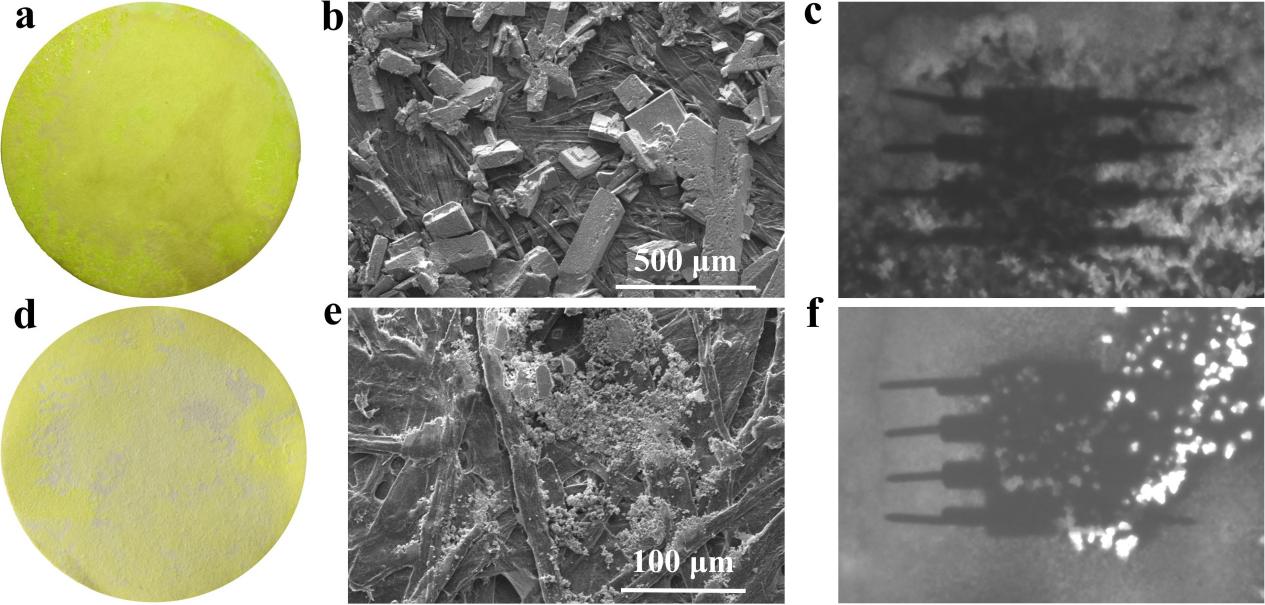


**Figure S14.** Optical images of Compound-I@paper scintillation screen prepared at (a) 20℃, and (d) 60℃. Top-view SEM image of Compound-I@paper scintillation screen prepared at (b) 20℃, and (e) 60℃. X-ray imaging based on Compound-I@paper scintillation screen prepared at (c) 20℃, and (f) 60℃.

**Note:** **Compound-I**@paper films prepared at 20℃ and 60℃ exhibit the poor quality (Figure S14a, S14d). When performing X-ray imaging, the flexible scintillation screens have a lower X-ray imaging quality (Figure S14c, S14f).


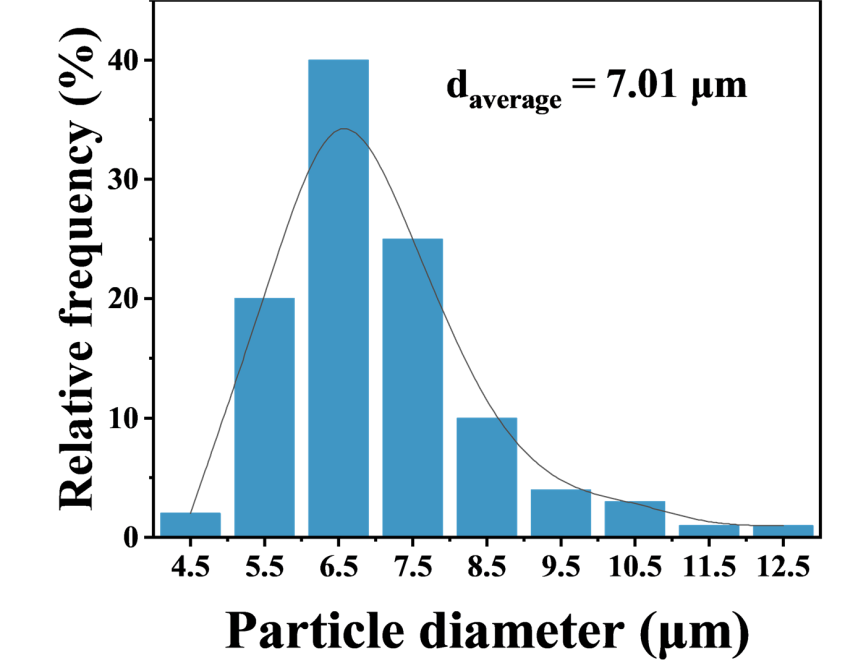


**Figure S15.** The diameter distribution diagram of **Compound-I** microcrystals in **Compound-I**@paper.


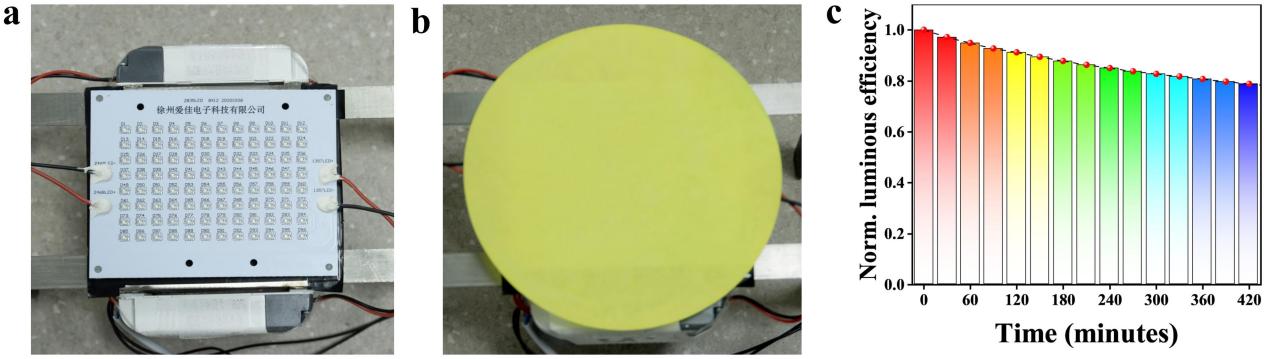


**Figure S16.** (a) Optical image of 460 nm LED array. (b) Optical image of the as-fabricated WLED based on **Compound-I**@paper film. (c) Operational stability of WLED.


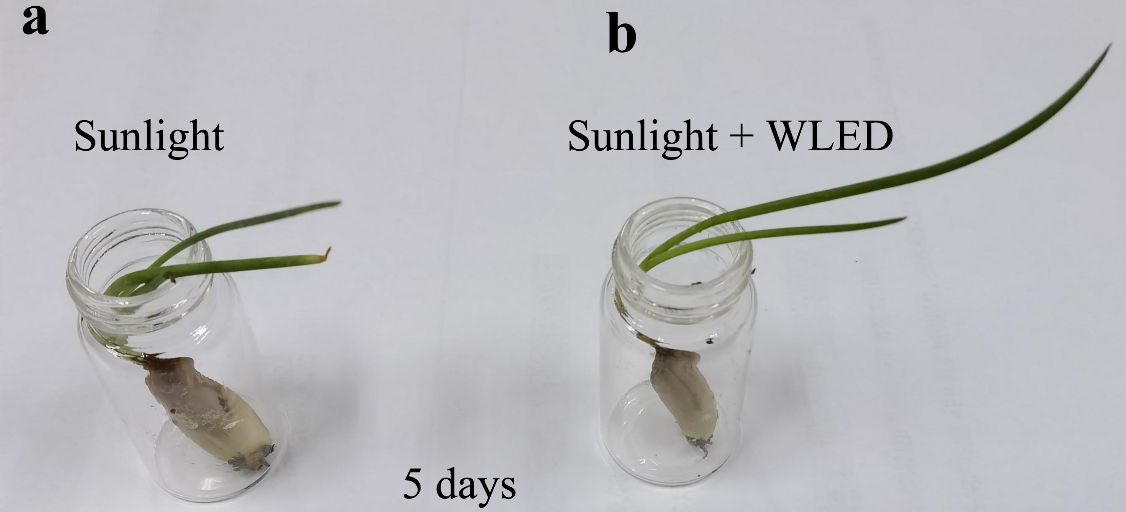


**Figure S17.** (a) Photos of garlic sprouts under different lighting conditions after 5 days. (a) Under sunlight irradiation. (b) Under sunlight and WLED irradiation.


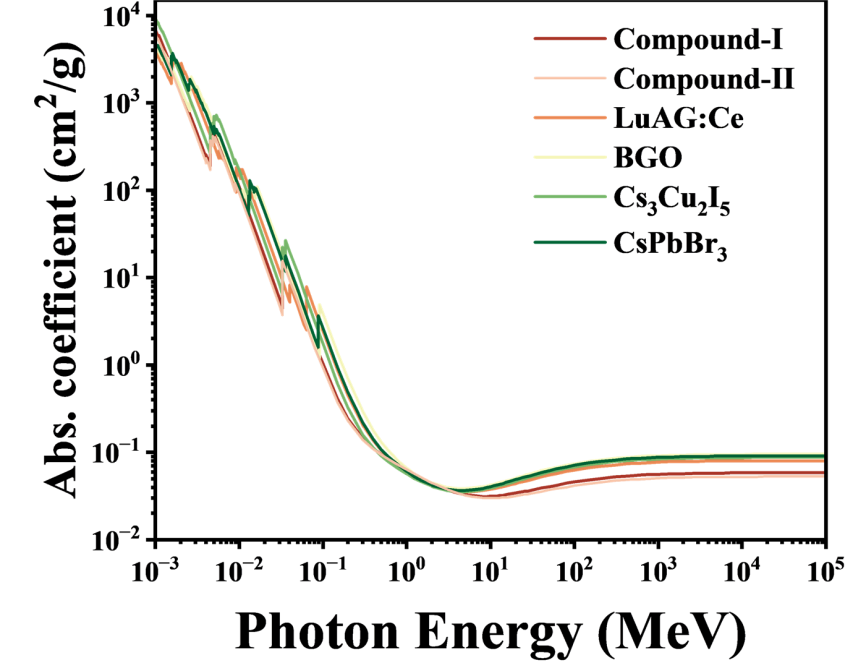


**Figure S18.** X-ray absorption coefficients of **Compound-I**, **Compound-II**, LuAG:Ce, BGO, CsPbBr_3_, and CsCu_2_I_5_.


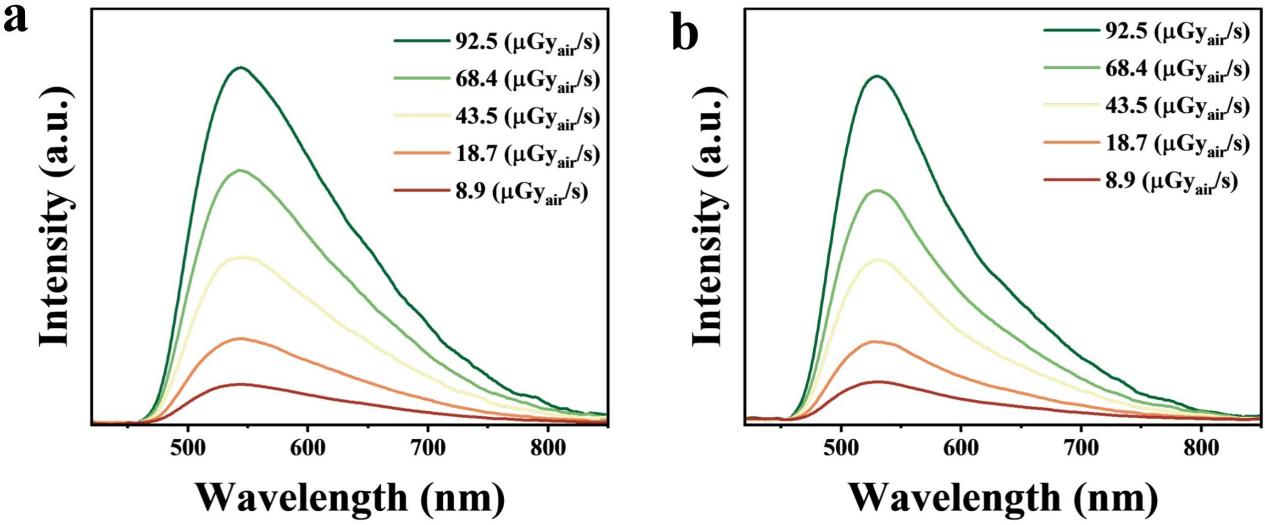


**Figure S19.** RL spectra of (a) **Compound-I** and (b) **Compound-II** under X-ray excitation at different dose rates (8.9-92.5 mGy_air_/s).


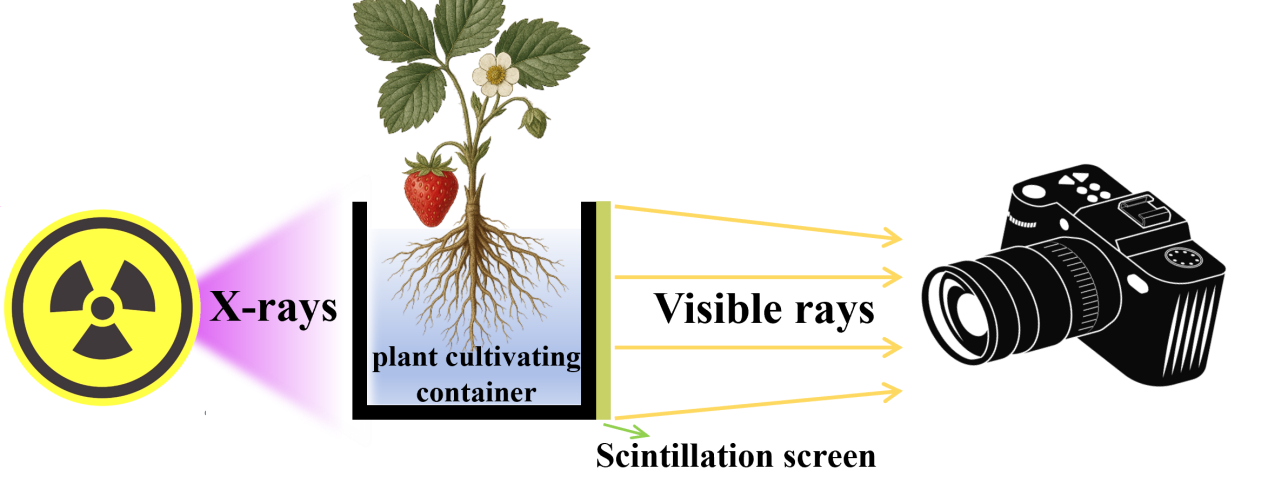


**Figure S20.** Plant root X-ray imaging system.


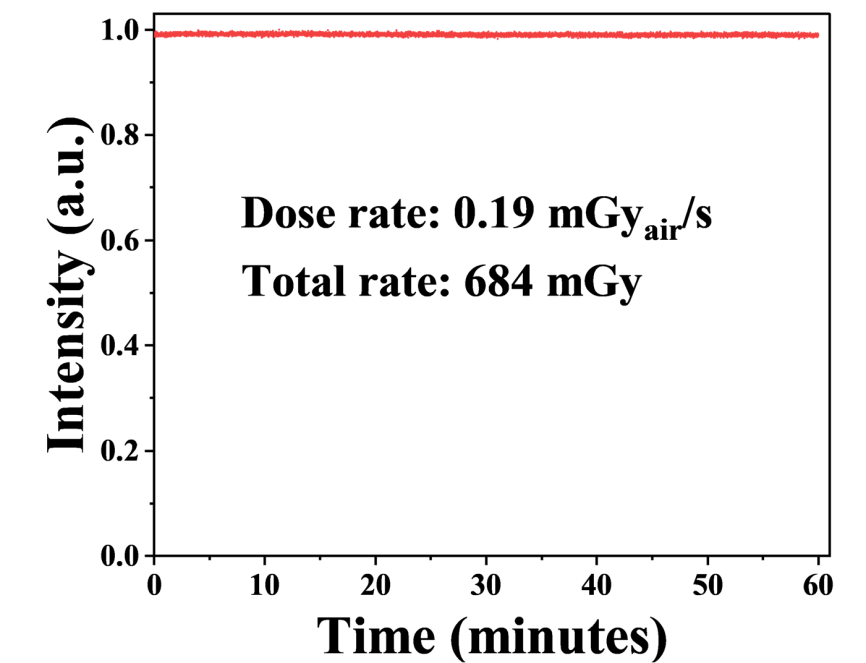


**Figure S21.** RL intensity of **Compound-I**@paper film upon X-ray continuous irradiation (X-ray dose rate, 0.19 mGy_air_/s).


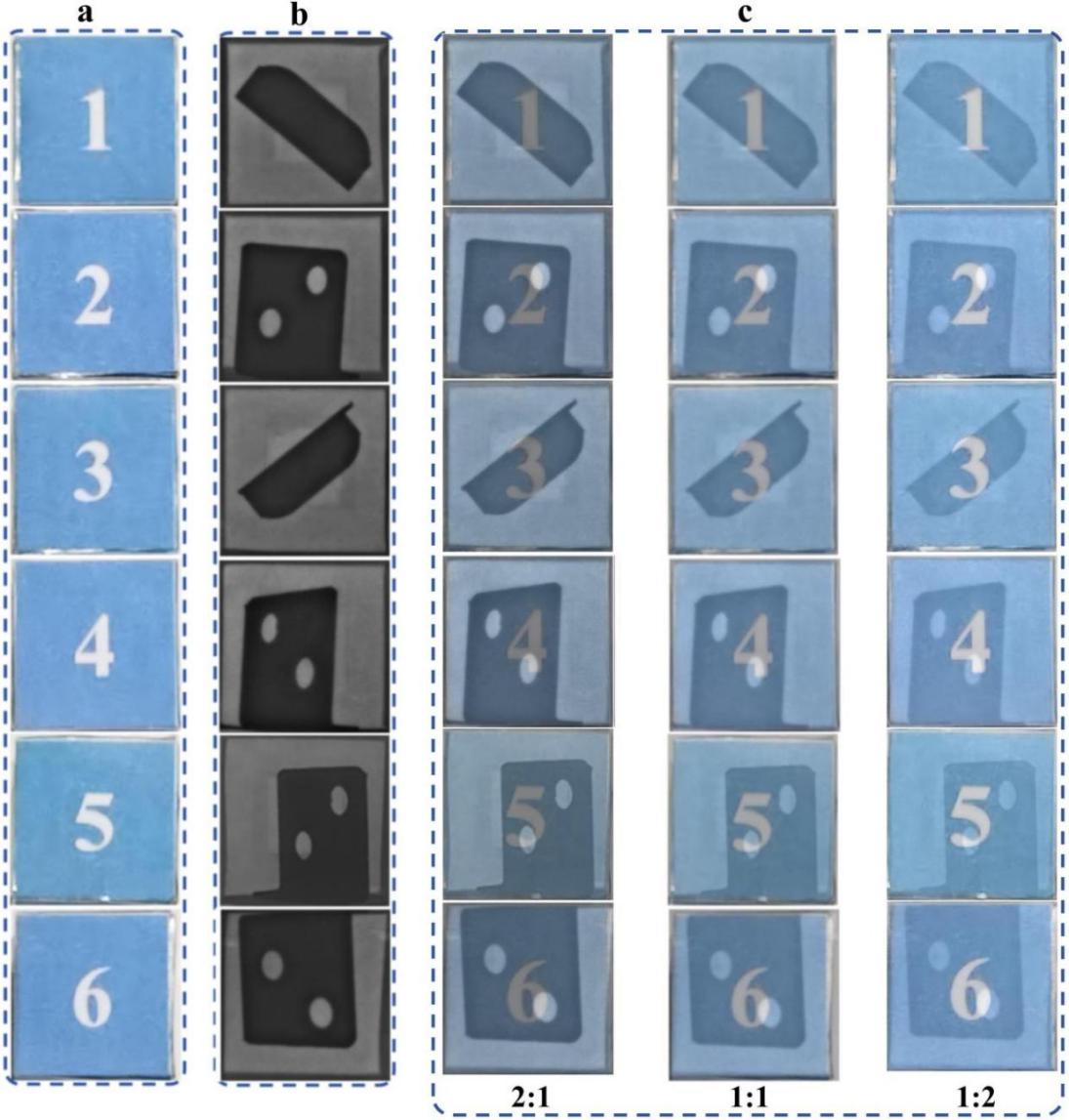


**Figure S22.** (a) Multi-angle white light images. (b) Multi-angle X-ray images. (c) White light and X-ray image fusion with different weights coefficients.


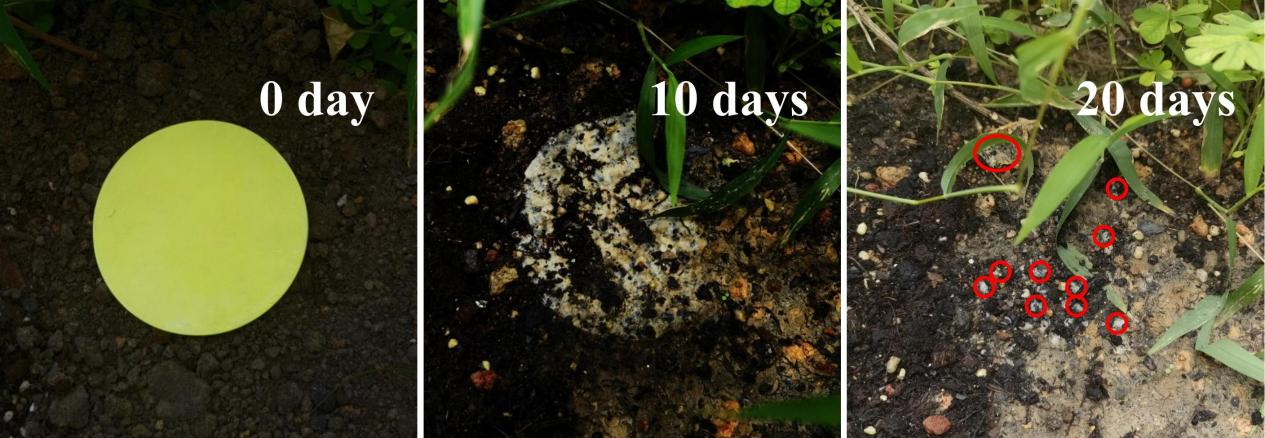


**Figure S23.** Biodegradability test of **Compound-I**@paper film in natural soil environment.

**References**

[1] S. Zhou, Y. Chen, K. Li, X. Liu, T. Zhang, W. Shen, M. Li, L. Zhou, R. He, *Chem. Sci.* **2023**, *14*, 5415.

[2] P. Fu, S. Geng, R. Mi, R. Wu, G. Zheng, B. Su, Z. Xia, G. Niu, J. Tang, Z. Xiao, *ENERGY Environ. Mater.* **2024**, *7*, e12518.

[3] K. Chen, B. Chen, L. Xie, X. Li, X. Chen, N. Lv, K. Zheng, Z. Liu, H. Pi, Z. Lin, A. L. Rogach, *Adv. Funct. Mater.* **2024**, *34*, 2310561.

[4] H. Shao, X. Wu, J. Zhu, W. Xu, L. Xu, B. Dong, J. Hu, B. Dong, X. Bai, H. Cui, H. Song, *Chem. Eng. J.* **2021**, *413*, 127415.

[5] X. Liu, Y. Li, L. Zhou, M. Li, Y. Zhou, R. He, *Adv. Opt. Mater.* **2022**, *10*, 2200944.

[6] Y. Xu, X. Hu, H. Tang, Q. Hu, S. Wang, T. Chen, X. Zhang, W. Jiang, L. Wang, W. Jiang, *Nanoscale* **2023**, *15*, 631.

[7] L. Xie, Z. Liu, H. Yang, K. Chen, N. Lv, H. Pi, X. Chen, X. Li, Z. Liu, S. Li, Z. Wang, Y. Wang, B. Chen, *Adv. Opt. Mater.* **2024**, *12*, 2401050.

[8] P. Du, L. Luo, W. Cheng, *J. Am. Ceram. Soc.* **2020**, *103*, 1149.

[9] Q. Wang, T.-C. Liu, W. Jiang, P.-Y. Xuan, X.-Y. Li, F. Guan, X.-W. Lei, Z.-H. Jing, X.-W. Kong, *Mater. Today Chem.* **2024**, *40*, 102263.

[10] L. Jiang, L. Wu, H. Sun, H. Yin, Q. Zou, J. Deng, R. Li, H. Ye, J. Li, *Chem. Eng. J.* **2024**, *494*, 153060.

[11] Q. Wang, S. K. Abkenar, M. Cirignano, H. Yu, W. Wu, G. Divitini, *J. Mater. Sci. Technol.* **2024**, *179*, 57.

[12] B. Yang, L. Yin, G. Niu, J. Yuan, K. Xue, Z. Tan, X. Miao, M. Niu, X. Du, H. Song, E. Lifshitz, J. Tang, *Adv. Mater.* **2019**, *31*, 1904711.

[13] S. Cao, J. Lai, Y. Wang, K. An, T. Jiang, M. Wu, P. Feng, P. He, X. Tang, *Laser Photonics Rev.* **2024**, *18*, 2400799.

[14] N. Lin, X. Wang, H.-Y. Zhang, K.-Q. Sun, L. Xiao, X.-Y. Zhang, C.-Y. Yue, L. Han, Z.-W. Chen, X.-W. Lei, *ACS Appl. Mater. Interfaces* **2024**, *16*, 41165.

[15] X. Hu, P. Yan, P. Ran, L. Lu, J. Leng, Y. M. Yang, X. Li, *J. Phys. Chem. Lett.* **2022**, *13*, 2862.

[16] Y.-H. Liu, N.-N. Wang, M.-P. Ren, X. Yan, Y.-F. Wu, C.-Y. Yue, X.-W. Lei, *ACS Appl. Mater. Interfaces* **2023**, *15*, 20219.

[17] H. Lv, W. Shao, H. Chen, G. Zhu, Y. Wang, Z. Zhang, H. Liang, *Adv. Sci.* **2025**, *12*, 2405995.

[18] Q. Zou, W. Yang, L. Wu, L. Jiang, S. Wang, L. Liu, R. Li, H. Ye, J. Li, *Chem. Eng. J.* **2025**, *506*, 159971.

[19] W. Wu, S. Lin, J.-X. Wang, Y. Xu, T. He, Y. Zhou, P. Yuan, P. Maity, I. Nadinov, S. Thomas, R. Huang, C. S. P. De Castro, J. Yin, H. N. Alshareef, O. M. Bakr, O. F. Mohammed, *Chem* **2025**, *11*, 102401.

[20] K. Han, J. Jin, B. Su, J. Qiao, Z. Xia, *Adv. Opt. Mater.* **2022**, *10*, 2200865.

[21] T. Xu, Y. Li, M. Nikl, R. Kucerkova, Z. Zhou, J. Chen, Y.-Y. Sun, G. Niu, J. Tang, Q. Wang, G. Ren, Y. Wu, *ACS Appl. Mater. Interfaces* **2022**, *14*, 14157.

[22] B. Tang, J. Jin, K. Han, T. Li, H. Zhang, X. Zhang, M. Molokeev, Y. Wang, Z. Xia, B. Lei, *Adv. Funct. Mater.* **2025**, *35*, 2500806.

[23] B. Li, K. Han, Y. Wang, Y. Sun, Z. Xia, Y. Xu, *Angew. Chem. Int. Ed.* **2025**, *64*, e202502440.

[24] J. Li, Q. Hu, J. Xiao, Z.-G. Yan, *J. Colloid Interface Sci.* **2024**, *671*, 725.

[25] Q. He, C. Zhou, L. Xu, S. Lee, X. Lin, J. Neu, M. Worku, M. Chaaban, B. Ma, *ACS Mater. Lett.* **2020**, *2*, 633.

[26] J. Jin, K. Han, Y. Hu, Z. Xia, *Adv. Opt. Mater.* **2023**, *11*, 2300330.

[27] J. Lai, P. Wang, B. Zheng, T. Xuan, D. Wu, Z. Wang, Y. Wang, W. Zhang, J. Du, P. He, K. An, X. Tang, *Adv. Opt. Mater.* **2024**, *12*, 2303297.

[28] Q. Peng, R. Cao, Q. Yang, Y. Si, J. Yuan, Y. Lei, Z. Wang, Q. Tang, K. Li, S. Zang, *Adv. Mater.* **2025**, *37*, 2504425.

[29] G. Kresse, J. Furthmüller, *Phys. Rev. B* **1996**, *54*, 11169.

[30] J. P. Perdew, K. Burke, M. Ernzerhof, *Phys. Rev. Lett.* **1996**, *77*, 3865.
